# p300/CBP inhibitor A-485 alleviates acute liver injury by regulating macrophage activation and polarization

**DOI:** 10.7150/thno.30707

**Published:** 2019-10-22

**Authors:** Jinjin Peng, Jiacheng Li, Jing Huang, Pan Xu, Heming Huang, Yanjun Liu, Liang Yu, Yaxi Yang, Bing Zhou, Hualiang Jiang, Kaixian Chen, Yongjun Dang, Yuanyuan Zhang, Cheng Luo, Guangming Li

**Affiliations:** 1 Department of Gastroenterology, Xinhua Hospital, School of Medicine, Shanghai Jiaotong University, 1665 Kongjiang Road, Shanghai 200092, China; 2 Drug Discovery and Design Center, State Key Laboratory of Drug Research, Shanghai Institute of Materia Medica, Chinese Academy of Sciences, 555 Zuchongzhi Road, Shanghai 201203, China; 3 University of Chinese Academy of Sciences, 19 Yuquan Road, Beijing 100049, China; 4 School of Pharmacy, Fujian Medical University, Fuzhou, China.; 5 School of Pharmacy, Guiyang University of Traditional Chinese Medicine, South Dong Qing Road, Huaxi District, Guizhou 550025, China.; 6 Key Laboratory of Metabolism and Molecular Medicine, the Ministry of Education, Department of Biochemistry and Molecular Biology, School of Basic Medical Sciences, Huashan Hospital, Fudan University, Shanghai 200032, China

**Keywords:** liver injury, inflammation, macrophages, epigenetics, acetyltransferase inhibitor

## Abstract

High morbidity and mortality are associated with acute liver injury (ALI) for which no effective targeted drugs or pharmacotherapies are available. Discovery of potential therapeutic targets as well as inhibitors that can alleviate ALI is imperative. As excessive inflammatory cytokines released by macrophages are a critical cause of liver injury, we aimed to find novel compounds that could inhibit macrophage expression of inflammatory cytokines and alleviate liver injury.

**Methods**: A high throughput assay was established to screen a small molecule inhibitor library of epigenetic targets. A highly selective catalytic p300/CBP inhibitor A-485 was identified as a potent hit *in vitro* and administrated to the lipopolysaccharide (LPS)/D-galactosamine (GalN)-induced mice *in vivo*. For *in vitro* analysis, RAW264.7 cells and primary BMDM cells exposed to LPS were co-incubated with A-485. A model of acute liver injury induced by LPS and GalN was used for evaluation of *in vivo* treatment efficacy.

**Results**: A-485 inhibited LPS-induced inflammatory cytokine expression in a concentration-dependent manner *in vitro*. Significantly, A-485 administration alleviated histopathological abnormalities, lowered plasma aminotransferases, and improved the survival rate in the LPS/GalN-stimulated mice. Integrative ChIP-Seq and transcriptome analysis in the ALI animal model and macrophages revealed that A-485 preferentially blocked transcriptional activation of a broad set of pathologic genes enriched in inflammation-related signaling networks. Significant inhibition of H3K27ac/H3K18ac at promoter regions of these pivotal inflammatory genes was observed, in line with their suppressed transcription after A-485 treatment. Reduced expression of these pathological pro-inflammatory genes resulted in a decrease in inflammatory pathway activation, M1 polarization as well as reduced leukocyte infiltration in ALI mouse model, which accounted for the protective effects of A-485 on liver injury.

**Conclusion**: Using a novel strategy targeting macrophage inflammatory activation and cytokine expression, we established a high-throughput screening assay to discover potential candidates for ALI treatment. We demonstrated that A-485, which targeted pathological inflammatory signaling networks at the level of chromatin, was pharmacologically effective *in vivo* and *in vitro*. Our study thus provided a novel target as well as a potential drug candidate for the treatment of liver injury and possibly for other acute inflammatory diseases.

## Introduction

Acute liver injury (ALI) is a common clinical disease caused by hepatotoxins originating from sepsis, side effects of drugs, alcohol abuse, metabolic syndrome, hepatitis virus, or bacterial infection. Despite considerable differences in their etiology, these conditions activate immune mechanisms that augment inflammation after the initial insult and may drive a lethal loss of hepatic function resulting from excessive death of hepatocytes 1. So far, there is no pathogenesis-specific clinical treatment for ALI. The available treatment for ALI/acute liver failure is mostly supportive and aims to protect hepatocytes and prevent complications caused by severe liver dysfunction 2. Therefore, there is an urgent need to identify specific targets and targeted drugs/chemo-therapies to treat ALI.

Cells of the monocyte-macrophage lineage constitute a significant component of the host defense system present in the liver 3-5. The pro-inflammatory response of macrophages has been implicated in the pathophysiology of ALI 3. There is also a large body of evidence showing that the inhibition of undesired inflammation regulated by macrophages is an attractive potential strategy in the treatment of liver inflammation and injury 4, 5. Macrophages are very plastic and adopt different phenotypes and transcriptional programs in response to the signals derived from the microenvironment. Macrophage function has traditionally been assigned as inflammatory or “M1 classically activated macrophage (CAM)” vs anti-inflammatory or “M2 alternatively activated macrophage (AAM)”. Excessive M1 macrophages in the injured liver are believed to be the source of pro-inflammatory cytokines which promote cell apoptosis and tissue damage.

Fine-tuned epigenetic machinery, regulating chromatin DNA or histones, has been reported to be involved in the macrophage activation as well as functional differentiation. HDAC3, a histone deacetylase, plays an important role in macrophage activation and inflammatory gene expression 6, 7. Similarly, DNMT3b knockdown has been reported to suppress adipose tissue macrophage activation and promote macrophage polarization to M2 phenotype 8. In recent years, the application of epigenetic modulators in inflammatory diseases has gained increasing attention. Several drugs targeting the epigenetic machinery, HDACi or DNMTi, as well as their combinations, have emerged as attractive potential therapies for the treatment of inflammation by modulating macrophages 6. Thus, small molecule compounds targeting epigenetic machinery might be an attractive pool for the discovery of potential drug candidates to inhibit macrophage activation. However, the lack of effective screening assays limited the discovery of novel epigenetic small molecule modulators of inflammatory macrophage activation and the exploration of the roles of epigenetic targets in this process. Previous studies were focused on testing the effects of HDACi or DNMTi on macrophage activation, therefore, discovery of new epigenetic modulators for manipulating macrophages is needed.

Epigenetic small molecule modulators remain a poorly exploited pool for the discovery of novel inhibitors of inflammatory macrophage activation. In this study, we aimed to identify novel targets as well as candidates for the treatment of ALI and other inflammatory diseases. We thus built an in-house epigenetic small molecule library containing 50 compounds, which were of high selectivity and potency for targeting crucial epigenetic proteins, and screened for novel epigenetic inhibitors of inflammatory macrophage activation. The high throughput screening identified A-485, a highly selective catalytic p300/CBP inhibitor, as a potent inhibitor of the excessive cytokine expression by macrophages. A-485 significantly alleviated liver injury relieving inflammation by epigenetically blocking transcriptional activation of a broad set of pathological genes, which were enriched in the inflammation-related signaling network.

## Materials and Methods

### Animal Experiments

Female C57BL/6J 10- to 12-week-old mice (specific pathogen-free), with body weights ranging from 20 to 22 g, were purchased from SIMM Animal Center (Shanghai, China). All mice were fed a standard laboratory diet and provided with free access to water. The animals were housed under standard laboratory conditions (21±2°C, 12-h light-dark cycle). All animal experiments were performed based on the institutional ethical guidelines on animal care and were approved by the Institute Animal Care and Use Committee at the Shanghai Institute of Materia Medica. Mice were randomly divided into three groups: NC group intraperitoneally injected with vehicle, ALI model group intraperitoneally injected with LPS (Escherichia coli, 0111:B4, Sigma, 2 mg/kg) and GalN (Sigma, 250 mg/kg) dissolved in PBS, and A-485-treated group (100mg/kg) intraperitoneally injected simultaneously with the treatment in the ALI model. After 4 hours of treatment, all mice were sacrificed and the liver tissues as well as blood were harvested. To evaluate the potential effects of A-485 on the survival rate in LPS/GalN-challenged mice, another set of mice were allocated to the same three groups described previously. Survival was assessed in the mice every 1 hour for 1 day.

### HE Staining, TUNEL Staining, and Immunohistochemical Analysis

Liver tissues were fixed in 4% paraformaldehyde overnight, embedded in paraffin, sectioned, and stained with hematoxylin and eosin (H&E; Wuhan goodbio G1005) or terminal deoxynucleotidyl transferase dUTP nick-end labeling (TUNEL; Roche 11684817910) according to the manufacturer's protocol. For immunohistochemistry, primary antibodies, which were directed against F4/80 (1:100, MAB5580, RD), Ly-6G (1:100, MAB1037-100, RD), CD86 (1:100, MAB0803, Novus), or CD206 (1:100, AF2535, RD), were incubated for 30 minutes at room temperature. Subsequently, the slides were further processed using corresponding secondary antibodies, followed by counter-staining with hematoxylin.

### Cell Culture

Cell lines RAW264.7 and L02 were cultured in Dulbecco's Modified Eagle Medium (DMEM) with 10% heat-inactivated fetal bovine serum (FBS, Gibco, Australia) using cell culturing plates (*In vitro* scientific). For stimulation, cells were treated with LPS (Escherichia coli, 055:B5, Sigma, 1 μg/mL) and/or A-485 (2.2 μM, 6.6 μM, 13.2 μM, 20 μM) for 4 h or 24 h. 293T cells were cultured in DMEM media with 10% FBS. All cells were cultured at 37°C, 5% CO_2_ with complete media and only exponentially growing cultures were used for assays.

Murine bone marrow derived macrophages (BMDM) were isolated from the tibia and femur of female C57BL/6J mice and were cultured in a sterile dish containing complete macrophage medium consisting of DMEM, 10% FBS, and 20 ng/ml M-CSF. For stimulation, cells were treated with LPS (Escherichia coli, 055:B5, Sigma, 1 μg/mL) and/or A-485 (2.2 μM, 6.6 μM, 13.2 μM, 20 μM) for 4 h or 24 h. Cells were cultured at 37°C, 5% CO_2_ with complete media.

### shRNA Construct and Transfection

*Ep300* shRNA sequences and a control shRNA were integrated into the lentiviral vector pLent-U6-GFP-Puro (Vigenebio, China), and the constructed shRNA- pLent-U6-GFP-Puro plasmid was transfected into 293T cells. The supernatant was collected and filtered at 48 and 72 h after transfection. RAW264.7 cells were infected with shRNA lentiviral particles in the presence of 10 μg/ml Polybrene. After two days of puromycin selection, RAW264.7 cells were collected for subsequent experiments. Primer sequences for *Ep300* knockdown are presented in Table S1.

### RNA-Seq Analysis

Total RNA isolated from RAW264.7 macrophages and mice liver tissues were used to prepare cDNA libraries that were subsequently sequenced on the Illumina HiSeq2000 using paired-end methods. The sequencing reads were mapped to mm10 by using STAR 2.5 and feature counts software was used to quantify gene expression 9. Differential gene expression analysis was performed by edgeR R package 10, 11. The p values were adjusted through the Benjamini & Hochberg method, and both 5% FDR cut-off and fold change greater than 1.5 were set as a threshold for significant genes. Differentially expressed genes were further analyzed by gene-annotation enrichment analysis using DAVID 6.8 bioinformatics platform. Network analysis was performed by using Cytoscape 12.

### ChIP-Seq and Motif-enrichment Analysis

ChIP analysis of H3K27ac and H3K18ac was performed as previously described 13, 14 using 1 × 10^7^ RAW264.7 cells. ChIP DNA was purified, and libraries were prepared with NEBNext® Ultra™II DNA Library Prep Kit from Illumina (NEB, E7645S). Raw reads were mapped to the mm10/GRCm38 Mus musculus genome with Bowtie (version 1.1.1) with the parameters -m 1 -k 1. ChIP-seq peaks were called by Model-based Analysis for ChIP-seq (MACS) (version 1.3.7.1) with the parameters -broad -nomodel -nolambda. We pooled the biological replicates together for each stage and performed the downstream analysis. Motif analysis was performed using HOMER 15, and target genes of TF were identified using ENCODE Transcription Factor Binding Site Profiles database, CHEA Transcription Factor Targets database and Cistrome Data Browser 16.

### Flow Cytometry Analysis

After different treatments, RAW264.7 cells and BMDM cells were resuspended in BD Pharmingen staining buffer (cat# 554657; BD Biosciences). Cells were incubated with FC block (cat# 553141; BD Biosciences) for 20 minutes. Subsequently, cells were washed and resuspended in 100 μl of BD Pharmingen staining buffer and incubated with PE Rat Anti-Mouse F4/80 (cat# 565410; BD Biosciences), FITC Rat Anti-CD11b (cat# 557396; BD Biosciences), PE-Cy^TM^7 Rat Anti-Mouse CD86 (cat# 560582; BD Biosciences), Alexa Flour® 647 Rat Anti-Mouse CD206 (cat# 565250; BD Biosciences) on ice for 30 minutes. Cells were washed three times with staining buffer and resuspended in staining buffer. Cells were centrifuged and resuspended in staining buffer for FACS analysis (FACS Celesta; BD Bioscience, San Jose, USA). FACS data analysis was performed using FLOWJO^TM^ Software.

### RNA Extraction and Quantitative RT-PCR

Total RNA extraction reagent (Vazyme, China) was used to isolate the total RNA from liver tissues and cells, and RNA was converted into cDNA with special cDNA synthesis kit (Vazyme, China) according to the manufacturer's protocol. Gene expression was measured using quantitative RT-PCR on Quant Studio 6 Flex Real-Time PCR system (ABI). Expression of target genes was normalized with *Gapdh* and calculated using ∆∆Ct method. The primer sequences are listed in Table S2.

### One Step RT-PCR

RAW264.7 cells were seeded in 96-well cell culturing plates (*In vitro* scientific) overnight and sequentially stimulated with LPS after 24h-pretreatment of compounds (20 μM) or LPS solely. Cells were harvested 4 hours after LPS stimulation with in-house lysis buffer. Cytolysis was then subjected to real-time qPCR using *TransScript*^®^ Green One-Step RT-qPCR Supermix (AQ211, Transgene).

### Western Blotting

Total protein was obtained from frozen liver and cultured cells. After quantification, samples were separated by SDS-PAGE and transferred onto a nitrocellulose membrane (Millipore, Temecula, CA, USA). Target protein bands were visualized using the enhanced chemiluminescence method in a ChemiScope3400 imaging system using ECL substrate (Clinx). Primary antibodies used are listed in Table S3.

### Biochemical Analysis and Cytokine Measurement

Serum alanine aminotransferase (ALT), aspartate aminotransferase (AST) and total bilirubin (T-Bil) were assessed using a Hitachi 7020 automatic analyzer (Hitachi, Tokyo, Japan). Cytokines were quantitatively measured using Elisa kits (Thermo, 88-7064), according to manufacturer protocol.

### ELISA

The concentration of IL-6, TNF-α, and IL-1β in liver tissues or culture supernatants were analyzed using ELISA kits (No. EMIL6RA, 88-7324-22, BMS6002; Thermo Scientific) according to the manufacturer's instructions. All samples were analyzed in duplicates.

### Statistical analysis

All numerical results were expressed as the mean ± SD and represented data from a minimum of three independent experiments. A two-tailed unpaired *t*-test was used to analyze differences between two groups. All analyses were performed using GraphPad Prism 7.0 statistical software (GraphPad Software, Inc., La Jolla, CA, USA). The level of statistical significance was set at P<0.05.

## Results

### High-throughput screening identified novel compounds that inhibited macrophages *Il1β* production in response to LPS stimulation

Up to now, high-throughput screening method has not been used to identify small-molecule compounds, which can inhibit the inflammatory response, for pharmacological applications in treating ALI or other inflammation-related metabolic disorders. We, therefore, sought to identify small molecule compounds that could inhibit inflammatory cytokine release by macrophages by setting up a screening assay. Our high-throughput assay was based on one-step qPCR for detecting cytokine* Il1β* mRNA expression as a readout for macrophage activation, because IL-1β stands at the center of the inflammatory response and is one of the major initiators of liver injury 17-19. Antibody blocking the IL-1 type I receptor (IL-1RI) to alleviate ALI 19 and some compounds hindering IL-1β functions have been reported to have potent anti-inflammatory effects 20.

**Figure 1:**
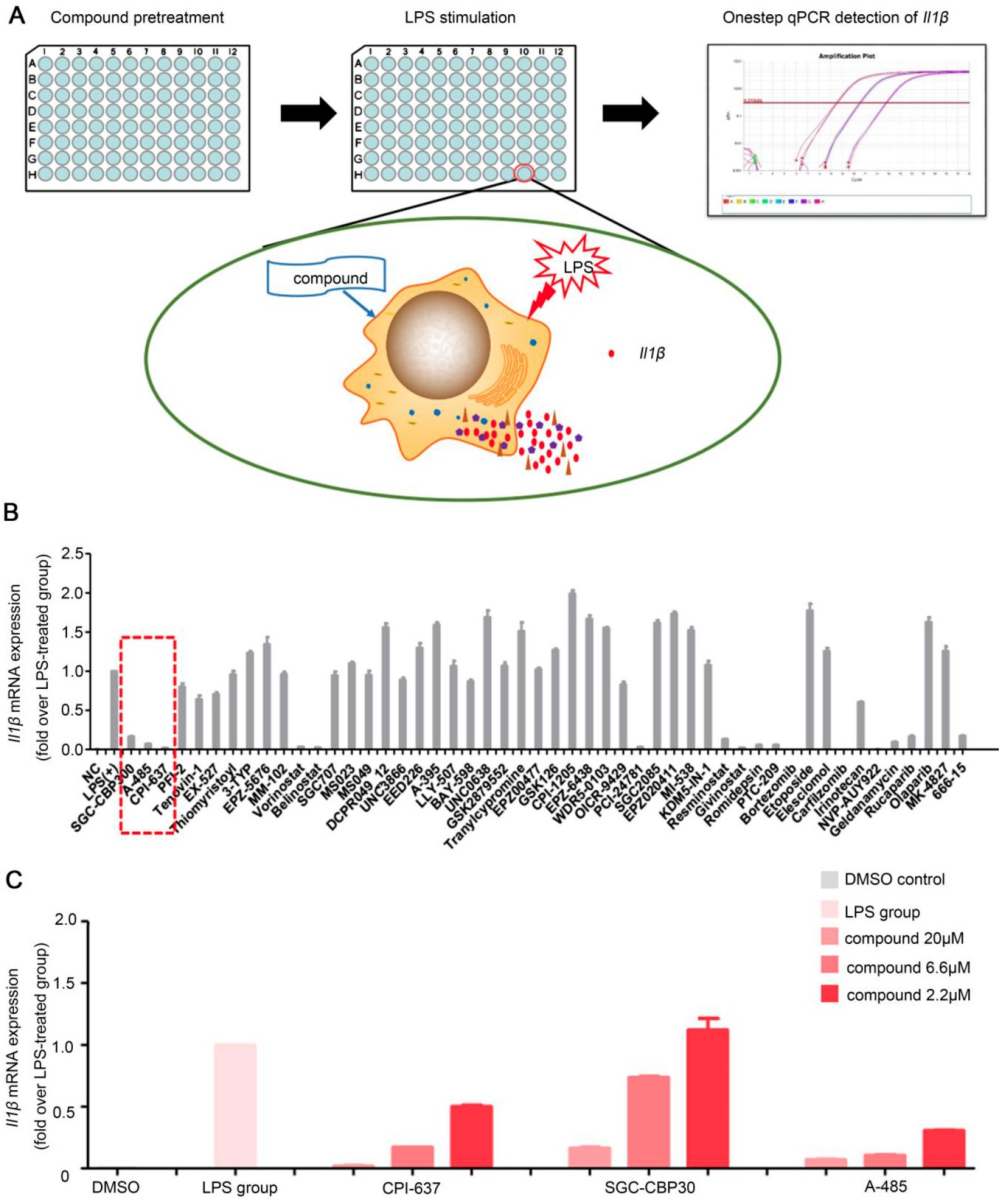
** One-step RT-qPCR identified a selective catalytic p300/CBP inhibitor A-485 that decreased LPS-stimulated macrophage *Il1β* production. (A)** Flow chart of the high-throughput screening assay for small-molecule inhibitors of macrophage activation and cytokine expression. **(B)** Results of high-throughput screening using a library containing 50 small-molecule probes. Inhibitors highlighted by dashed line in red are categorized as p300/CBP inhibitors. **(C)** Effects of p300/CBP inhibitors. SGC-CBP30, CPI-673, and A-485 are inhibitors of p300/CBP, inhibiting the bromodomain (SGC-CBP30 and CPI-673) or the histone acetyltransferase domain (A-485). The RT-qPCR data were normalized to a reference gene, *Gapdh*, and are shown as mean ± SD based on three independent experiments.

RAW264.7 cells were seeded in 96-well plates and stimulated by LPS in the presence of each compounds in the library (Figure 1A). Subsequently, the library containing 50 molecule probes targeting epigenetic proteins was screened to find small molecule compounds able to inhibit *Il1β* expression (Table S4). In total, we identified more than 10 hits including belinostat, vorinostat and PCI-24781 (HDAC inhibitor); MI503 (Menin-MLL inhibitor); PTC-209 (BMI-1 inhibitor); 666-15 (CREB inhibitor); SGC-CBP30 and CPI-673 (p300/CBP bromodomain inhibitors); A-485 (p300/CBP HAT domain inhibitor); rucaparib (PARP inhibitor); bortezomib and carfilzomib (proteasome inhibitors) (Figure 1B). These compounds could significantly inhibit the mRNA expression of *Il1β* induced by LPS stimulation in RAW264.7 cells. The inhibitory effect of vorinostat 21, belinostat 22, PARP inhibitor 23, proteasome inhibitors bortezomib 24 and carfilzomib 25 on macrophage activation and excessive cytokine release has been described before and validated high efficiency of our high-throughput screening assay to find effective molecules. We noticed that all there inhibitors targeting the transcriptional coactivator p300/CBP/CREB complex, SGC-CBP30 (CBP bromodomain inhibitor) 26, CPI-673 (p300/CBP bromodomain inhibitor) 22 and A-485 (p300/CBP histone acetyltransferase domain inhibitor) 27 showed striking inhibitory effects. A-485, a newly reported highly selective catalytic p300/CBP inhibitor, showed the most potent inhibitory effects (Figure 1C). Whether p300 could serve as a potential target and p300 inhibitors could inhibit macrophage activation and alleviate ALI has not been studied previously. We, therefore, focused on A-485 to investigate its potential role in the treatment of acute liver injury.

### Inhibition of p300 suppressed macrophage inflammatory responses

**Figure 2:**
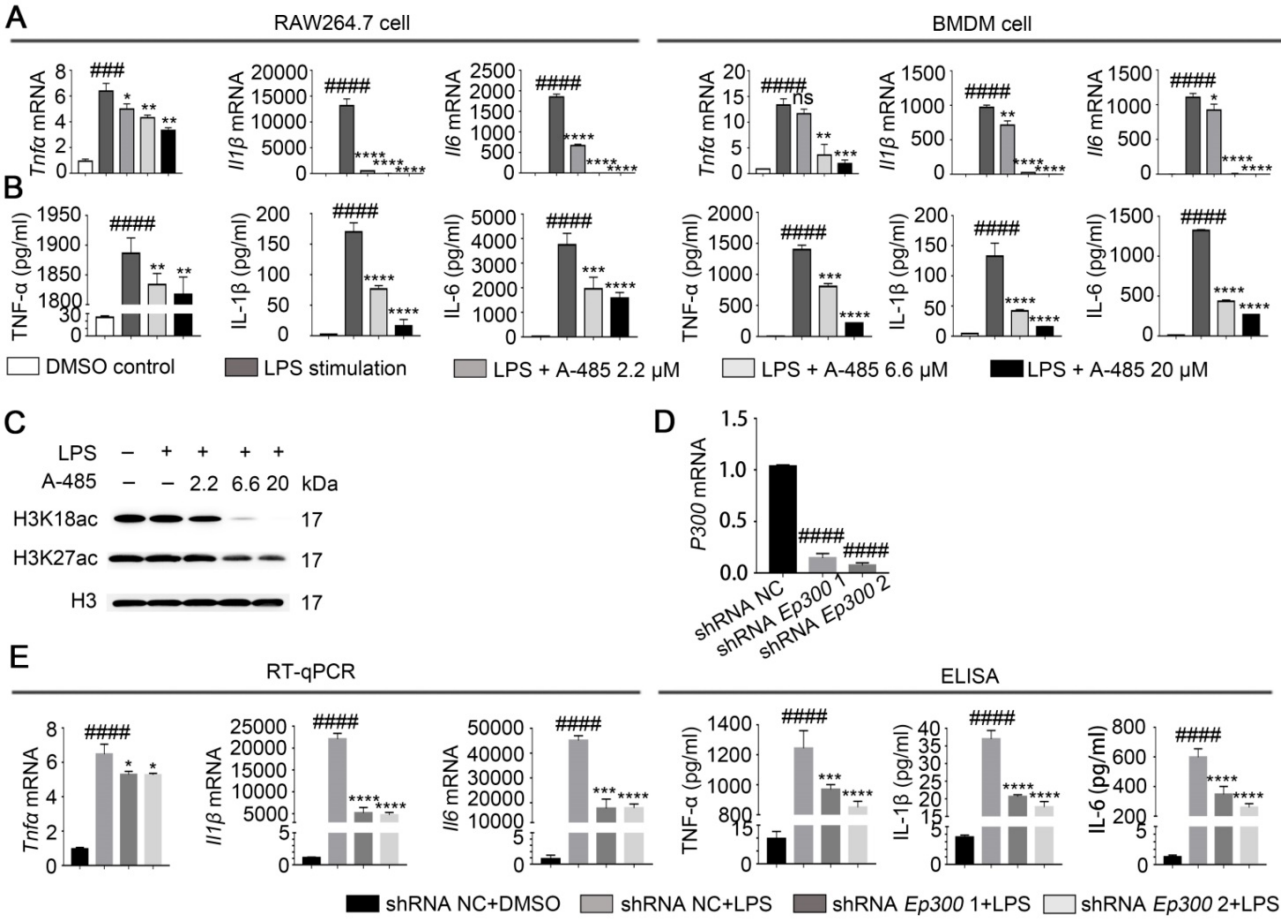
** A-485 inhibited inflammatory responses through inhibition of the catalytic function of p300. (A)** RAW264.7 and BMDM cells were treated with LPS and A-485 for 4 h, after which the *Tnfα*, *Il1β*, and *Il6* mRNAs were quantified by RT-qPCR analysis. **(B)** RAW264.7 and BMDM cells were treated with LPS and A-485 for 24 h. The TNF-α, IL-1β and IL-6 concentrations in culture supernatants were determined by ELISA. **(C)** Western blot analysis of H3K27 and H3K18 acetylation in LPS-challenged RAW264.7 cells. **(D)** The knockdown efficiency of shRNAs targeting *Ep300* in RAW264.7 cells at the mRNA level**. (E)** RT-qPCR analysis of *Tnfα*, *Il1β* and *Il6* mRNA in *Ep300* knockdown and DMSO control RAW264.7 cells stimulated with LPS for 4 h **(E, Left)**. TNF-α, IL-1β, and IL-6 concentrations in culture supernatants were detected by ELISA after stimulating with LPS for 24 h **(E, Right)**. (n=3) Data are shown as mean ± SD. ns* P*>0.05, *P<0.05, **P<0.01, ***P<0.001 and ****P<0.0001 vs LPS group, ###* P*<0.001 and ####*P*<0.0001 vs control group.

We next evaluated the *in vitro* effect of A-485 on LPS-induced cytokine expression, which is indicative of macrophage inflammatory activation. IL-6, TNF-α, and IL-1β are recognized as representative inflammatory mediators, and their expression levels are positively correlated with the severity of the inflammatory response 28. We cultured RAW264.7 and BMDM cells in the presence or absence of A-485 and treated them with LPS (1μg/mL) for 4 or 24 h. RT-qPCR and ELISA were used to detect the mRNA expression levels in the cells or protein secretion levels in the supernatant, respectively, of *Il6*, *Tnfα*, and *Il1β*. Data showed that RAW264.7 cells stimulated with LPS showed a significant increase in the mRNA and protein expression levels of *Il6*, *Tnfα* and *Il1β*. However, co-treatment with A-485 abrogated the LPS-induced transcription and expression of these cytokines in a concentration-dependent manner (Figure 2A, 2B; left panel). Similarly, A-485 treatment also impaired LPS-induced pro-inflammatory gene expression at both mRNA and protein levels in BMDM cells (Figure 2A, 2B; right panel). When we shortened the co-treatment time to 4 h, A-485 similarly decreased protein secretion of pro-inflammatory cytokines (TNF-α, IL-1β, and IL-6) stimulated by LPS (Figure S1A). We also examined the effect of A-485 on hepatocytes under LPS challenge. The mRNA expression of *Il1β*, *Il6*, and *Tnfα* in hepatocytes (L02 cells) remained unchanged in response to either LPS stimulation or A-485 administration (Figure S1B). Furthermore, A-485 treatment showed little cytotoxicity in both macrophages and hepatocytes (Figure S2). These data demonstrated that the p300/CBP HAT inhibitor had cell-specific inhibitory effects on inflammatory gene expression *in vitro*, and also suggested that macrophages were a major cell target of A-485.

A-485 was reported to specifically inhibit p300/CBP-catalyzed acetylation of histone H3 lysine 27 (H3K27) and lysine 18 (H3K18) sites. Western blotting showed reduced H3K27 and H3K18 acetylation in RAW264.7 cells treated with A-485, thus confirming that the anti-inflammatory effect of A-485 was associated with enzymatic inhibition of its target p300/CBP (Figure 2C, S3). Quantifications of the western blot data was showed in Figure S3. To further prove that the anti-inflammatory effect of A-485 was due to targeted inhibition of p300, we tested the effect of *Ep300* knockdown on cytokine expression and secretion in RAW264.7 cells, which were infected with lentivirus carrying short hairpin RNA (shRNA) targeting *Ep300* gene or non-targeting shRNA. Knocking down efficiency was confirmed by RT-qPCR 48 h post infection (Figure 2D). The knockdown and control cells were stimulated with LPS, and mRNA and protein levels of *Il6*, *Tnfα*, and* Il1β* were quantified by RT-qPCR as well as ELISA. Consistent with the anti-inflammatory effects of A-485, *Ep300* knockdown significantly inhibited the expression of these cytokines (Figure 2E). The pharmacological inhibitor A-485 showed stronger effect than *Ep300* knockdown, perhaps due to the fact that A-485 simultaneously targeted both p300 and CBP or that *Ep300* knockdown efficiency was less potent compared with chemical inhibition. Taken together, these results suggested that A-485 potently inhibited pro-inflammatory gene expression and protein secretion through inhibition of the catalytic function of p300.

### A-485 exerted protective effects on a LPS/GalN-induced ALI model in mice

**Figure 3:**
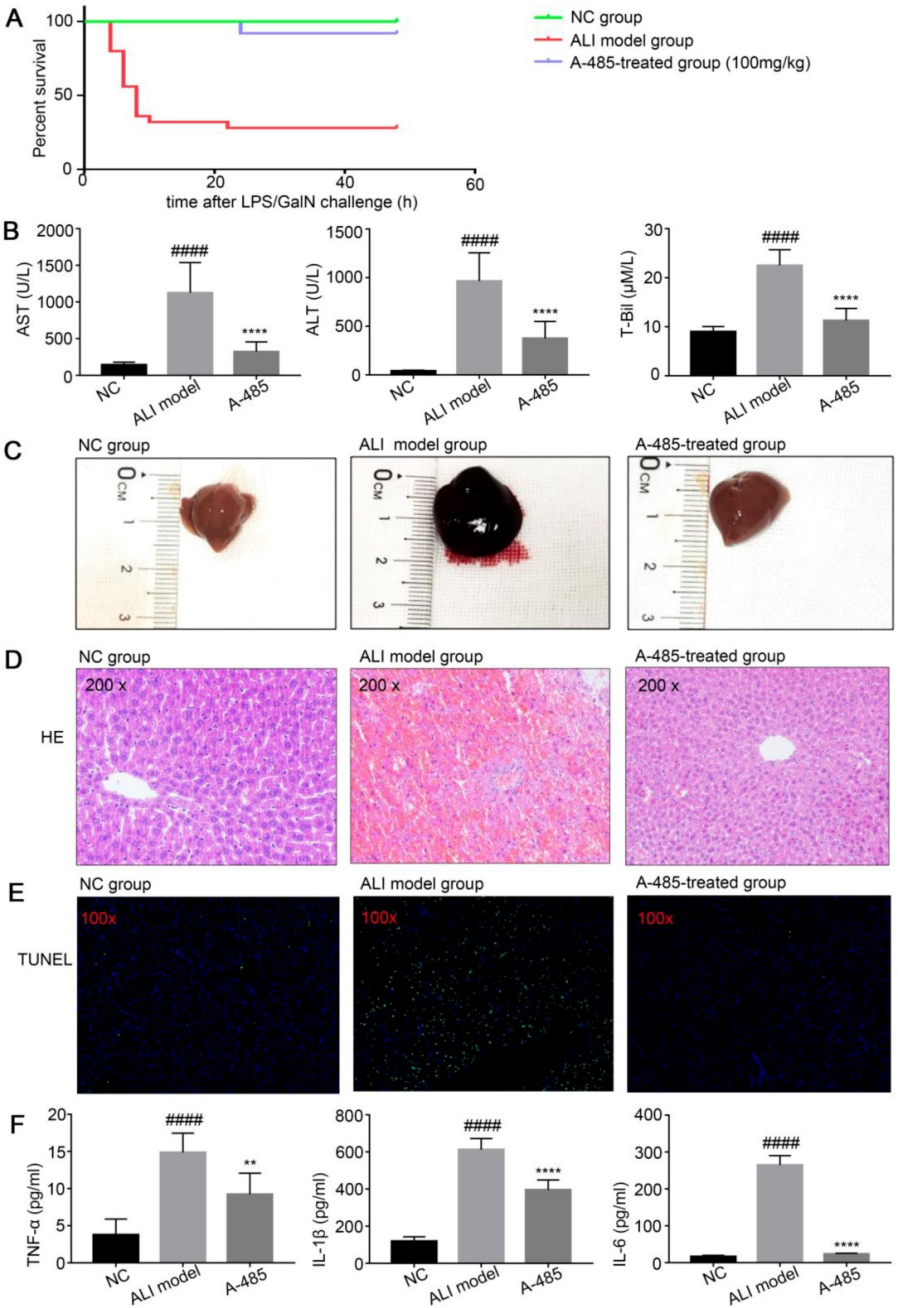
** A-485 exerted protective effects on the LPS/GalN-induced ALI model in mice. (A)** Survival curves for mice from the NC group (n=15), ALI model group (n=25), and A-485-treated group (n=25). **(B)** Serum ALT, AST, and T-Bil in mice from the NC group, ALI model group, and A-485-treated group (n=15). **(C)** Morphological changes of livers in mice from the indicated groups. **(D and E)** Histological examination of liver tissues. H&E **(D)** and TUNEL **(E)** staining of liver tissues of mice from the indicated groups. **(F)** ELISA detected the TNF-α, IL-1β, and IL-6 concentrations in liver tissues (n=6). Data are shown as mean ± SD. ****P<0.0001 vs ALI model group, ####*P*<0.0001 vs control group.

Pro-inflammation cytokines have been reported to promote hepatic injury in ALI mice 29, 30. We examined whether A-485 treatment could protect mice from LPS/GalN-induced ALI by inhibiting cytokine expression in mice. After the mice were injected with LPS/GalN, they were observed for 24 h to determine the survival rate. Consistent with the previous studies, we observed markedly increased incidence of lethality in LPS/GalN-induced ALI mice. Compared with LPS/GalN injected ALI animals, 24 h survival rate of the ALI group increased significantly upon co-treatment with A-485 at 100mg/kg (ALI group: 28% vs A-485: 92%, p<0.0001, Figure 3A). The levels of serum ALT, AST and T-Bil, three important indicators of liver function, were markedly higher at 4 h after LPS/GalN exposure compared to the control group indicating severe liver injury induced by LPS/GalN. Co-treatment with A-485 significantly reduced the content of serum ALT, AST and T-Bil (Figure 3B).

Furthermore, the liver morphology was apparently improved to the naked eye. Compared with the control group, the liver appearance of mice in the ALI group was significantly deepened in color and enlarged in volume; this abnormal macroscopic morphology was significantly alleviated when mice were co-treated with A-485 (Figure 3C). Also, HE staining of liver tissues in ALI mice showed severe histological abnormalities, including liver tissue structure destruction, hepatocyte necrosis, congestion and inflammatory cell infiltration. These effects were clearly ameliorated in the A-485-treated group (Figure 3D). Similarly, TUNEL staining showed increased hepatocyte apoptosis in the liver tissues of ALI mice while A-485 co-treatment could significantly reduce apoptotic cells in the liver (Figure 3E). Consistently, significant cleavage and activation of Caspase3 protein were observed in the liver tissues of the ALI group, and this process was obviously inhibited after A-485 intervention (Figure S4). These data collectively suggested that mice co-treated with A-485 significantly reduced the severity of liver injury.

We noticed that H3K27ac and H3K18ac levels were markedly higher in ALI liver compared with control liver which suggested the involvement of acetylation in ALI pathogenesis. A-485 treatment reduced H3K27ac and H3K18ac to the level comparable to the control group indicating targeted inhibition of p300/CBP (Figure S5). We next tested whether A-485 inhibited pro-inflammatory cytokine expression *in vivo*. Compared with the control group, the expression of pro-inflammatory mediators (IL-1β, IL-6, and TNF-α) increase obviously in the ALI model group. Also, ELISA assays revealed that A-485 treatment significantly reduced these cytokine levels in the supernatant, which is consistent with the *in vitro* results (Figure 3F). Collectively, these results established reliable evidence that A-485 inhibited inflammatory cytokine expression and hepatocyte apoptosis, reduced the structural and functional damage of liver tissue, and promoted survival of mice with ALI.

### A-485 blocked transcriptional activation of a broad set of pathological genes enriched in inflammatory cytokines and chemokines

**Figure 4:**
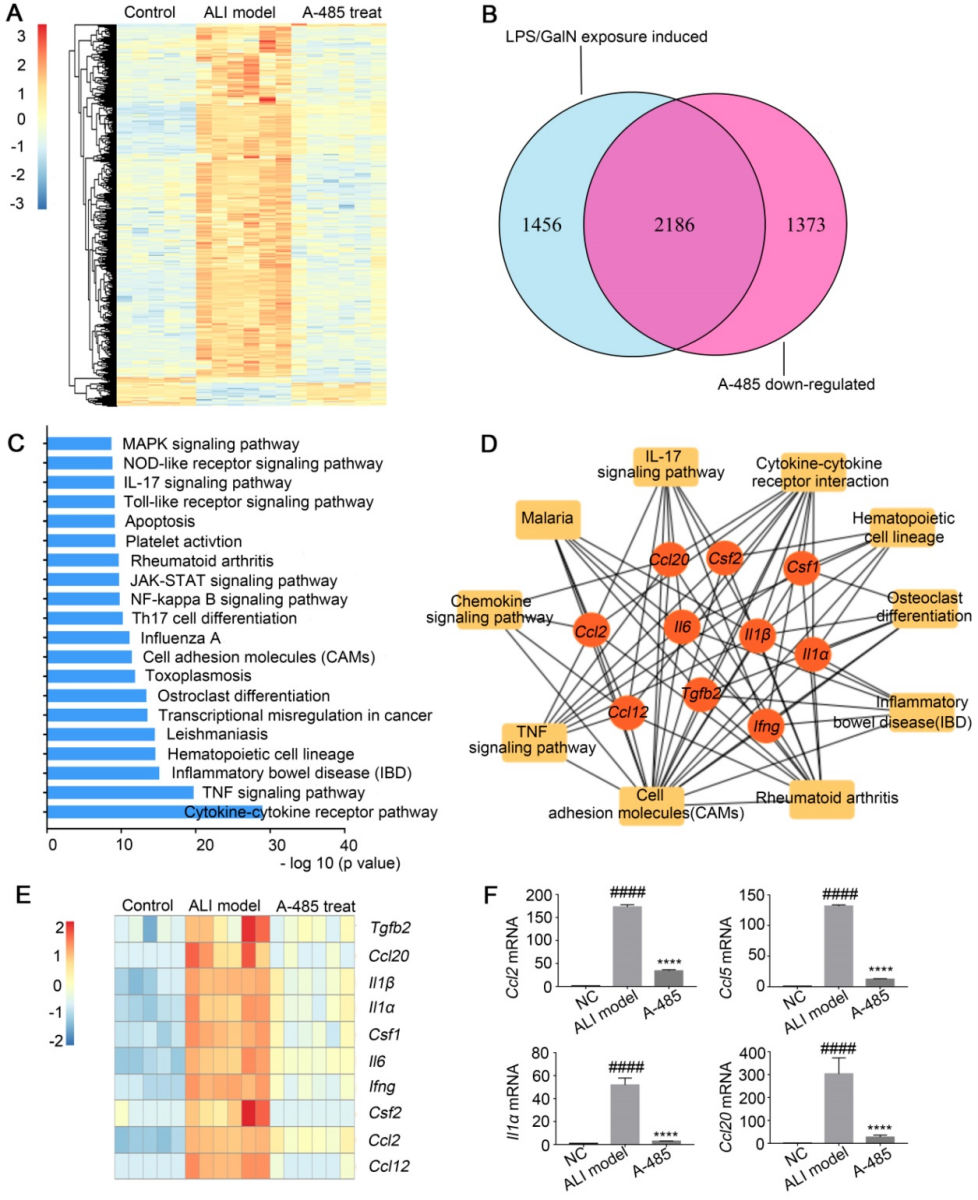
** A-485 regulated inflammatory gene expression *in vivo* as determined by RNA sequencing. (A-D)** RNA-seq analysis was performed on liver tissues extracted from mice in the control (n=5), ALI model (n=6), and A-485-treated groups (n=6). **(A)** The heat map of genes with adjusted P value <0.05, and absolute value of log2 fold-change >1.5. (B) Venn diagram showing the overlap between the gene set of LPS/GalN-induced upregulated genes and the gene set subsequently downregulated by A-485 *in vivo*. **(C)** KEGG pathway analysis using differentially expressed genes between the LPS/GalN-exposed ALI group and the control group, showing that the most significantly enriched pathways are related to the inflammatory response. **(D)** Visualization of inflammation network and top 10 hub genes. Inflammatory gene network was constructed by mapping genes from the top 10 most significantly affected pathways to the PPI network using Maximal Clique Centrality method. **(E)** Heat map of top 10 hub genes. **(F)** RT-qPCR analysis was performed to validate the repression of hub genes by A-485 (n=5). Data are shown as mean ± SD. ****P<0.0001 vs ALI model group, ####*P*<0.0001 vs control group.

To explore the mechanism underlying the substantial improvement in survival as well as liver structure and function in ALI by A-485 treatment, RNA-seq analysis was employed to compare the gene expression profiles of liver tissues from control mice and LPS/GalN-challenged mice with or without A-485 treatment. Unsupervised hierarchical clustering was applied to explore the gene expression profile among the three groups. As shown in Figure 4A, while the clustering pattern from the LPS/GalN exposure group was highly different (Figure 4A), whereas clustering patterns of gene expression between the control group and A-485-treated group were quite similar, reflecting broad suppression of pathologic state. LPS/GalN exposure induced vast gene expression changes. There was significant upregulation of 3642 genes upon LPS/GalN stimulation, among which the most noteworthy feature was the systematic production of inflammatory cytokines and chemokines. In A-485-treated group, 60% (2186) of genes induced by LPS/GalN challenge were reversed which was consistent with the significant improvement in the ALI phenotype (Figure 4B). The KEGG pathway analysis performed using differentially expressed genes between the LPS/GalN-exposed ALI group and the control group showed that predominantly affected pathways were associated with inflammation and apoptosis (Figure 4C), which were significantly suppressed by A-485 treatment.

Network analysis was further conducted by mapping genes from the top 10 most significantly A-485-affected pathways to the PPI network. The results revealed cross-links between inflammatory pathways of which the hub genes showed top 10% connectivity in the pro-inflammatory network (Top 10 hub genes are shown as Figure 4D and 4E, the full list of hub genes is shown in Table S5). These hub genes stood at the convergence points of the prominently affected pathways by A-485 treatment. Most hub genes were cytokines and chemokines with crucial functions in the inflammatory response and have been reported to be central for ALI pathogenesis, such as *Il1β*, *Il1α*,* Il6*, *Ccl2*, and *Ccl12*. A-485 repressed expression of these hub genes *in vivo* (Figure 4E, 4F) which could further inhibit the cascade signal transduction of inflammatory response. In summary, these results suggested the significant alleviation of ALI by A-485 can be mainly attributed to diminishing the pathological gene expression related to inflammatory activation.

**Figure 5:**
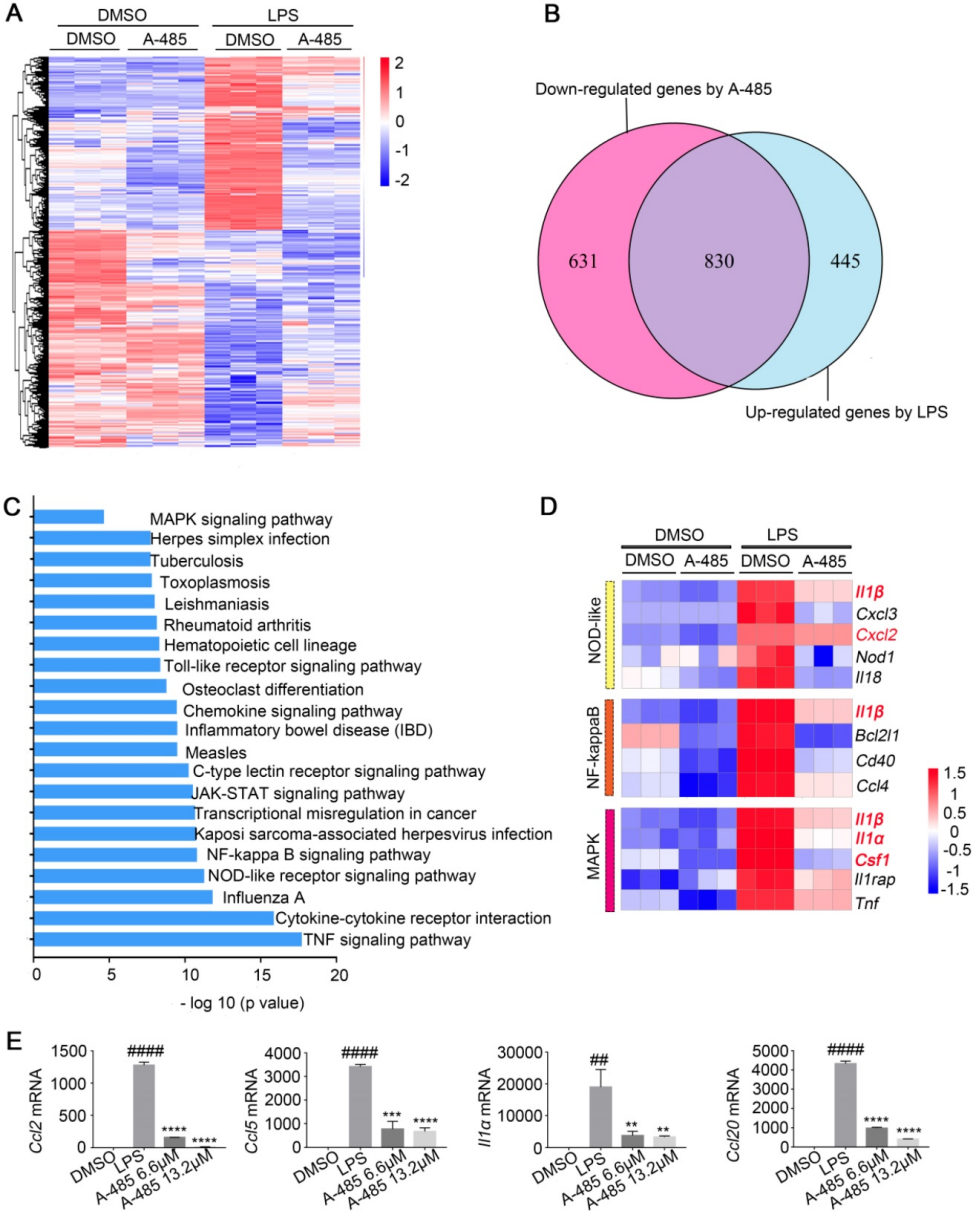
** A-485 regulated inflammatory gene expression in RAW264.7 cells as determined by RNA sequencing. (A-E)** RNA-seq analysis was performed using RAW264.7 cells treated with DMSO or LPS (1 μg/ml) for 4 h, or treated with LPS and A-485 (13.2 μM) for 4 h (n=3). **(A)** Heat map of genes with adjusted P value <0.05, and absolute value of log2 fold-change >1.5. **(B)** Venn diagram showing the overlap between the LPS-induced genes and the genes whose activation was reduced by A-485. **(C)** KEGG pathway analysis using differentially expressed genes between the LPS-exposed and A-485-treated groups. **(D)** Heat map of representative key genes in the NF-κB signaling pathway, NOD-like receptor signaling pathway, and MAPK signaling pathway. Hub genes are highlighted in red. **(E)** RT-qPCR analysis was performed to validate the repression of hub genes by A-485 (n=3). Data are shown as mean ± SD. **P<0.01, ***P<0.001 and ****P<0.0001 vs ALI model group; ##*P*<0.01, and ####*P*<0.0001 vs DMSO group.

We next investigated the molecular mechanism by which A-485 ameliorated the inflammatory response in the macrophage model challenged by LPS. To gain a more global view of gene expression changes after A-485 treatment, we carried out RNA-seq analysis on RAW264.7 in the following groups: DMSO control group, A-485 6.6 μM control group, A-485 13.2 μM control group, LPS stimulation + DMSO group, LPS stimulation + A-485 6.6 μM group, and LPS stimulation + A-485 13.2 μM group (Figure S6). Four of these groups are shown in Figure 5A. When comparing the DMSO control group and LPS stimulation + DMSO group, a large number of genes were up-regulated upon LPS stimulation, most of which reversed to the level close to the DMSO control group after A-485 treatment (Figure 5A). Of the 1275 LPS-induced genes, 830 (65%) showed significantly repressed expression when cells were treated with A-485 (13.2 μM) (Figure 5B) which implied that the most of LPS-induced inflammatory transcriptional process was compromised after A-485 administration. The inhibitory effects of A-485 were specific since A-485 had no broad effect on rescuing genes down-regulated after LPS stimulation, consistent with the primary function of p300 as a transcriptional activator. By analyzing the effect of A-485 on genes which were repressed upon LPS stimulation, we found the overall impact of A-485 administration to be relatively small, with only 185 (11 %) of 1630 LPS-repressed genes partially or completely rescued (Figure S7). Furthermore, comparison between the DMSO control and A-485 group revealed that A-485 showed mild effects on basal expression of the LPS-induced gene expression (Figure 5A). The contribution of A-485 to relieve LPS-induced repression was relatively marginal. Thus, A-485 inhibited a broad but specific set of pathological genes related to inflammation.

Further gene-annotation enrichment analysis revealed that most prominent KEGG pathways affected in LPS-activated macrophages were inflammation-related and were broadly inhibited by A-485 treatment. These results were consistent with RNA-seq analysis *in vivo* (Figure 4C, 5C). Also, most of pro-inflammatory cytokines and chemokines previously identified as hub genes in ALI mouse model (Figure 4D) were inhibited by A-485 administration (Figure 5D). Among the enriched KEGG pathways, TNF, Cytokine-cytokine receptor interaction, NOD-like receptor, NF-kappaB, Toll-like receptor and MAPK signaling pathways ranked among the top significantly influenced pathways following A-485 treatment (Figure 5C). RT-qPCR analysis verified that A-485 administration inhibited expression of the hub genes in macrophages as well as in liver tissues (Figure 4F, 5E). Together, these data indicated that A-485 dampened pro-inflammatory state in response to LPS by suppressing a broad subset of pathological genes, especially pro-inflammatory cytokines and chemokines.

### A-485 suppressed acetylation of H3K27 and H3K18 at pro-inflammatory gene promoters

**Figure 6:**
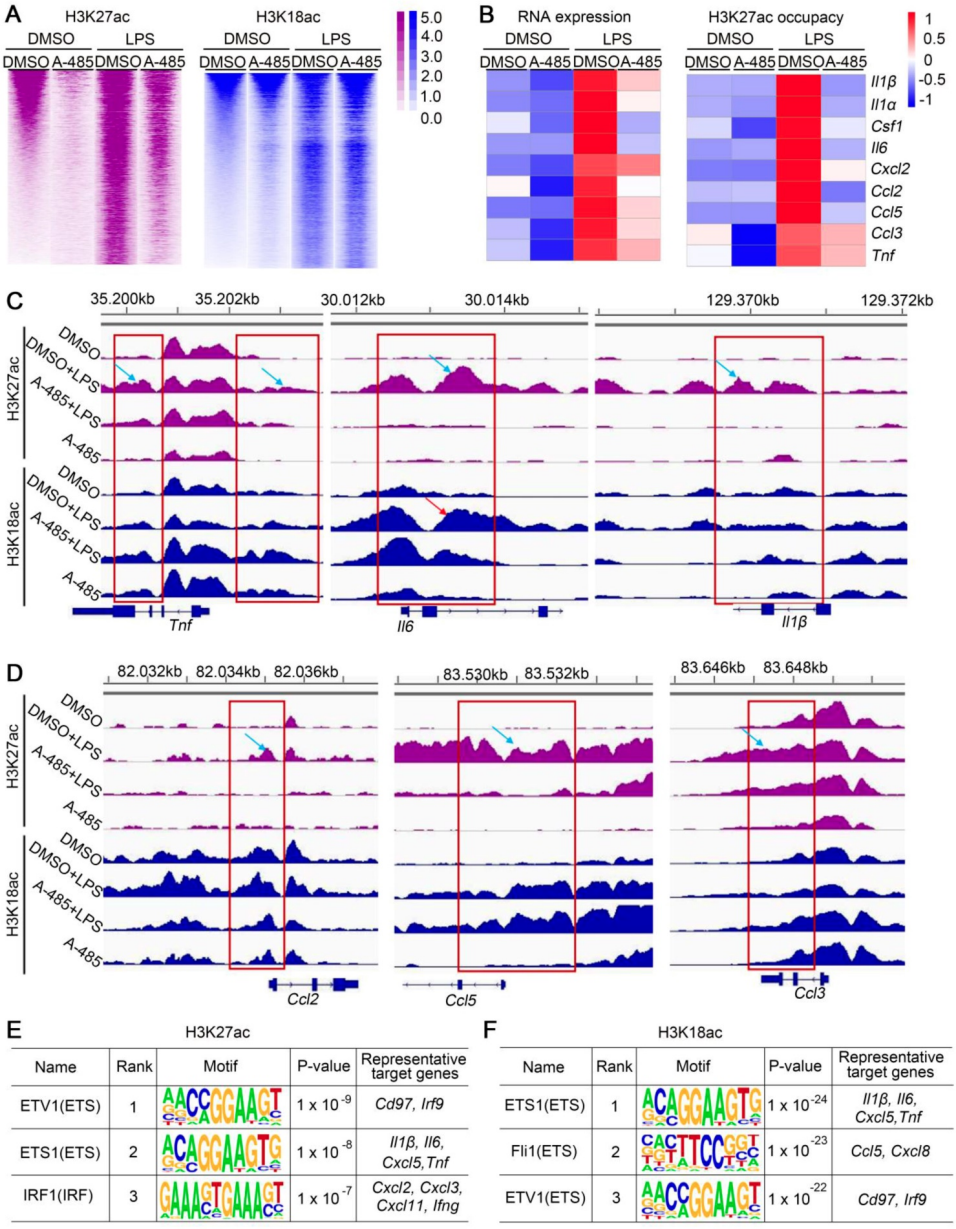
** A-485 suppressed H3K27ac and/or H3K18ac density at promoter regions of pivotal pro-inflammatory genes. (A)** Heat map displaying ChIP-Seq signal enrichment changes in H3K27ac (left) and H3K18ac (right) between indicated groups.** (B)** Heat map of gene expression (left) and H3K27ac occupancy (right) of representative inflammatory genes.** (C-D)** Read density of H3K27ac and H3K18ac at pivotal cytokine and chemokine genes (such as *Tnf*, *Il6*, *Il1β*, *Ccl2*, *Ccl5*, and *Ccl3*) in macrophages from four indicated groups. Gene tracks were visualized using Integrative Genomics Viewer. **(E-F)** Top 3 most enriched A-485 affected motifs in H3K27ac ChIP-seq data **(E)** and H3K18ac ChIP-seq data** (F)**. Representative target genes for these TF are identified using ENCODE Transcription Factor Binding Site Profiles database, CHEA Transcription Factor Targets database and Cistrome Data Browser.

As a specific p300/CBP HAT domain inhibitor with high selectivity, A-485 reversed the increased H3K27ac/H3K18ac level in response to LPS/GalN challenge *in vivo* and LPS stimulation *in vitro*. Modification of H3K27ac and H3K18ac plays a key role in the activation of gene expression. RNA-seq analysis in ALI model mice as well as in macrophages showed that A-485 effectively suppressed inflammatory gene expression. To test whether repressed gene expression was due to decreased H3K27ac/H3K18ac by A-485, we performed ChIP-seq to explore genome-wide H3K18ac/H3K27ac alternations, and correlated changes in H3K27ac/H3K18ac with changes in gene expression after A-485 treatment. LPS stimulation altered the H3K27ac and H3K18ac distribution state. H3K27ac and H3K18ac density increased significantly when compared to the DMSO-treated group (Figure 6A), indicating that the transcriptional activation of LPS-induced inflammatory genes was dependent on H3K27ac and H3K18ac. A-485 treatment reduced H3K27ac notably while H3K18ac to a much less extent (Figure 6A, S8).

Integrating and comparing the changes of H3K27ac and H3K18ac with the transcriptomic changes induced by A-485 treatment revealed the direct mechanism by which A-485 alleviated inflammation response. We performed integrative ChIP-Seq and transcriptome analysis to identify the subset of LPS-induced genes, whose expression was under the direct control of acetylation modification at promoter regions mediated by p300/CBP. Among the 1275 LPS-inducible genes identified in RNA-seq analysis, 311 showed elevated H3K27ac or H3K18ac at their promotors upon LPS stimulation (Figure S9A). Notably, 176 of these genes (56.59%) were specifically repressed by A-485 at both transcriptional and epigenetic/chromatin levels (Figure S9B). Most importantly, A-485 treatment reduced mRNA expression as well as H3K27ac/H3K18ac deposition at promoter regions of most pivotal hub genes, which were at the convergence point of A-485-inhibited inflammation-related pathways and acted as master regulators of inflammation, such as *Il1β*, *Il1α*, *Il6*, *Ccl5*, and *Ccl2* (Figure 6B). Gene tracks showing H3K27ac and H3K18ac peaks at *Tnf*, *Il6*, *Il1β*, *Ccl2*, *Ccl5*, and *Ccl3* promoters are displayed in Figure 6C and 6D. LPS stimulation caused significantly increased H3K27ac and H3K18ac peaks at the promoters of *Tnf*, *Il6*, *Il1β*, *Ccl2*, *Ccl5*, and *Ccl3*, implying enhanced transcriptional activity of inflammatory genes (Figure 6C, 6D). The H3K27ac and H3K18ac density at these inflammatory genes induced by LPS was significantly suppressed by A-485 (Figure 6C, 6D).

Motif enrichment analysis was further performed to reveal potential transcriptional factors whose transcriptional activation of these pathological genes were associated with H3K27ac/H3K18ac and were affected by A-485. Firstly, genomic locations with increased H3K27ac or H3K18ac level after LPS administration (LPS group & DMSO control group) were used to identify TF binging motifs associated with increased H3K27ac or H3K18ac after LPS stimulation. As expected, enriched motifs associated with increased H3K27ac or H3K18ac after LPS stimulation are mainly binding motifs for transcription factors which play crucial roles in inflammation response (Table S6, S7). These results suggested that binding of these important transcription factors to target gene promoter in response to LPS couples with H3K27ac and H3K18ac deposition at the region around the TF binding motifs to facilitate transcription. Next, motif enrichment analysis was performed in genomic locations with reduced H3K27ac or H3K18ac level by A-485 treatment (LPS + A-485 group & LPS group) (Table S6, S7). Notably, for H3K27ac ChIP-seq data, among top 30 most enriched LPS-affected motifs (associated with increased H3K27ac after LPS stimulation), 20 (66.67%) of them were identified as A-485 affected motifs (with decreased H3K27ac around the TF binding motif after A-485 treatment) (Table S6). Similar results were obtained when H3K18ac ChIP-seq data were used for motif analysis. 21 (70%) of top 30 LPS-affected motifs (associated with increased H3K18ac after LPS stimulation) were identified as A-485 affected motifs (with decreased H3K18ac around the TF binding motif after A-485 treatment) (Table S7). Among these identified A-485 affected motifs (with increased H3K27ac/H3K18ac level in response to LPS, and decreased level after A-485 treatment), binging motifs for ETV1, ETS1, IRF1 were the three most enriched in H3K27ac ChIP-seq data, while ETS1, Fli1 and ETV1 were the three most enriched in H3K18ac ChIP-seq data. ETS and IRF families play important roles in regulating pro-inflammatory genes 31-33, and target genes of ETS1, Fli1, ETV1, and IRF1 contained many crucial pro-inflammatory cytokines and chemokines (Figure 6E, 6F). Taken together, motif-enrichment analysis revealed that A-485 treatment inhibited H3K27ac/H3K18ac deposition around the TF binding region of the pathological inflammatory genes, indicating reduced transcriptional activation of pathologic genes by these crucial transcriptional factors.

Taken together, A-485 suppressed expression of a broad set of pathological genes, especially pivotal pro-inflammatory genes, by decreasing H3K27ac/H3K18ac deposition at their promoters and hindering their transcriptional activation by crucial TFs, restraining the inflammatory cascades.

### A-485 impaired activation of key pro-inflammatory pathways and reduced infiltration of macrophages and neutrophils *in vivo*

**Figure 7:**
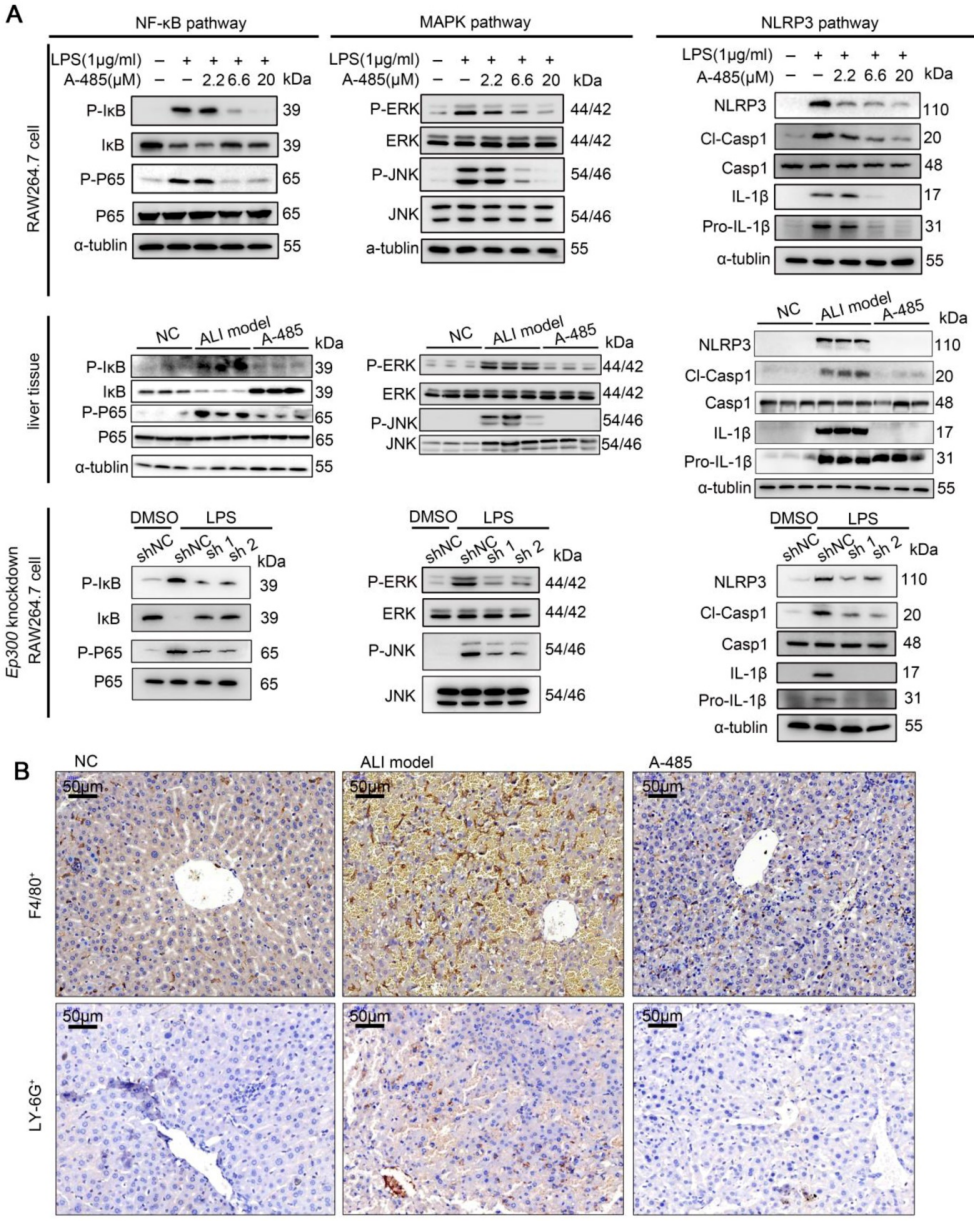
** A-485 inhibited activation of inflammatory pathways and inflammatory cell infiltration. (A)** Western blotting was performed to validate the activation of NF-κB, MAPK and NLRP3 signaling pathways in RAW264.7 cells, liver tissues, and *Ep300* knockdown RAW264.7 cells.** (B)** Immunohistochemical staining of macrophages (F4/80^+^) and neutrophils (LY-6G^+^) within liver tissue.

The results of ChIP-seq together with RNA-seq showed A-485 treatment epigenetically silenced hub genes encoding pro-inflammatory cytokines and chemokines at the chromatin level, including *Tnfα*, *Il6*, and *Il1β*. These genes directly have been reported to activate NF-κB, MAPK and NLRP3 signaling pathways 34-37, triggering inflammatory cascade and augmented detrimental inflammation. These signaling pathways have been reported to be crucial for ALI pathogenesis 38-40. As shown by our transcriptomic analysis, inhibition of pro-inflammatory genes by A-485 probably led to repression of these signaling pathways. We thus next examined the effect of A-485 on the activation of NF-κB, MAPK, and NLRP3 signaling in LPS-stimulated macrophages and ALI liver tissues. Western blotting showed that treatment of A-485 impaired the activation of NF-κB, MAPK, and NLRP3 pathways in macrophages (Figure 7A, top). Similar results were observed *in vivo* (Figure 7A, middle). Further, to demonstrate that inhibition of pathways was due to on-target instead of off-target effects, the influence of *Ep300* knockdown on pathways activation was assessed and was found to be similar to that of the compound treatment (Figure 7A, bottom). Quantifications of the western blot data was showed in Figure S10. These data demonstrated that A-485 strongly impaired the activation of inflammatory signaling pathways.

In the early stage of inflammation, secretion of pro-inflammatory cytokine TNF-α can drive macrophage polarization towards an inflammatory 'M1-like' phenotype 3, 41. Consistent with the massive inhibitory effects on cytokine expression and inflammatory pathway activation, A-485 inhibited macrophage M1 polarization in RAW264.7 and BMDM cells; this was evident by down-regulation of M1 markers induced by LPS, including *Il1β*,* Il6*, and *Tnfα*. In contrast, A-485 treatment up-regulated three M2 markers *Arg1*, *Ym1*, and *Cd301* (Figure S11A). Similar results were obtained in the liver tissue (Figure S11B). We performed flow cytometry to further confirm macrophage polarization by A-485 regulation. We marked F4/80^+^ CD11b^+^ macrophages, then used CD86 and CD206 antibodies to label M1 and M2 phenotypes, respectively. The data demonstrated that the M1 phenotype was increased after LPS stimulation of RAW264.7 and BMDM macrophages, and A-485 administration inhibited macrophage M1 polarization (Figure S11C, S11D). As for the M2 marker staining, there were no significant changes after LPS stimulation or A-485 administration. The results indicated that the pharmacological inhibitor, A-485, suppressed M1 polarization. Further, to demonstrate that inhibition of macrophage M1 polarization was due to on-target instead of off-target effects, the results of *Ep300* knockdown on macrophage polarization were assessed and were found to be similar to the A-485 treatment (Figure S11E).

During liver injury, bone marrow-derived monocytes accumulate in injured liver and differentiate into inflammatory macrophages. Chemokines, such as CCL2, CCL3, and CCL5, recruit inflammatory cells, leading to a vicious cycle of hyper-inflammation and further deteriorating liver injury. Our integrative ChIP-seq and transcriptome analysis revealed that A-485 inhibited transcriptional activation of key chemokines (*Ccl2*, *Ccl3*, and *Ccl5*) due to reduced H3K27ac/H3K18ac on their promoter regions (Figure 4F, 5E, and 6D). Immunohistochemical staining of liver tissues was performed to assess the effect of A-485 intervention on inhibition of chemokine-directed macrophages and neutrophil infiltration. Results showed that macrophages (marked by F4/80) and neutrophils (marked by LY-6G) increased in the liver of ALI model compared with the control group, and was significantly reduced after A-485 treatment (Figure 7B). More specific marker like CLEC4F (specific for Kupffer cells) and IBA-1 (both resident kupffer cells and infiltrated macrophage) was used to distinguish the resident Kupffer cells from infiltrated macrophages 42-44. The results showed that IBA-1^+^ cells increased in liver of ALI model mice compared with the control group, and was reduced after A-485 treatment (Figure S12). In contrast, the number of CLEC4F^+^ cells did not change significantly after LPS/D-Gal administration or after A-485 treatment (Figure S12). These data indicated that A-485 inhibited the infiltration of macrophages in ALI model, with little influence on proliferation of resident kupffer cells. These results indicated the pharmacological inhibition of the p300/CBP HAT effectively blocked the infiltration of inflammatory cells in ALI by inhibiting expression of chemokines. Collectively, these findings suggested that A-485 decreased the transcriptional activation of a broad set of pathological genes mainly encoding key cytokines and chemokine, resulting in impaired inflammatory signaling pathways, macrophage M1 polarization, as well as leukocyte infiltration.

## Discussion

As an inflammatory disorder with limited treatment options, ALI affects millions of people worldwide. In this study, we aimed to target macrophage activation and inflammatory cytokine expression and identified potential targets as well as novel compounds for the treatment of ALI. A high-throughput screening assay was established for the discovery of modulators of macrophage activation. Based on the potential role of epigenetic mechanisms in modulating macrophage function and reprogramming, an epigenetic inhibitor library was screened, identifying the highly selective catalytic p300/CBP inhibitor A-485 as a novel small molecule hit compound. Its inhibitory effects on ALI as well as on macrophage inflammatory activation were validated *in vitro* and *in vivo*.

The effects of p300/CBP inhibitors on ALI have not been studied previously. Herein, we report that A-485 alleviated liver injury and protected mice from LPS/GalN-induced death *in vivo*. The improvement in liver tissue structure and function as well as survival rate in ALI mice was strikingly significant. As shown by RNA-seq analysis, the inflammatory transcriptional program in ALI livers was significantly reduced and the majority of gene expression induced by LPS/GalN challenge was compromised after A-485 administration, indicating its potent protective effects *in vivo*. KEGG pathway analysis showed that predominantly affected pathways in ALI mice liver tissues were associated with inflammation, all of which were significantly inhibited after A-485 treatment. These data suggested A-485 was highly efficacious for inhibiting macrophage activation and alleviating liver injury. The inhibitory effects on inflammatory cytokine expression were also observed when *Ep300* was knocked down in macrophages, suggesting the anti-inflammatory effects of A-485 were due to target inhibition. Based on these observations, A-485 might be a highly efficacious anti-inflammatory small molecule and serve as a potential candidate for the treatment of inflammation-related liver diseases.

We also explored the mechanism underlying the anti-inflammatory effects of A-485 using integrative ChIP-Seq and transcriptome analysis *in vitro* and *in vivo*. RNA-seq analysis of liver tissues revealed that A-485 potently blocked the induction of pathological genes highly enriched for effectors of inflammation-related signaling pathways. Since inflammation is central to ALI pathogenesis and molecular mediators of inflammatory processes are intensely pursued therapeutic targets, p300 might represent a novel target for ALI intervention. Network analysis revealed the hub genes at the convergence of the top 10 most significant pathways inhibited by A-485, most of which were cytokines and chemokines crucial for ALI pathogenesis. In the macrophage model, RNA-seq and RT-qPCR validation suggested that A-485 treatment potently blocked a broad set of pathologic inflammation-related genes, including the hub genes. ChIP-seq analysis was carried out and compared with the transcriptional changes to reveal the direct mechanism by which A-485 alleviated inflammation response. A-485 treatment reduced mRNA expression as well as H3K27ac or H3K18ac deposition at promoters of inflammation-related genes, especially most of the hub genes. According to our motif-enrichment analysis, A-485 treatment reduced H3K27ac/H3K18ac deposition around the binding region of crucial inflammatory TFs in promoter region of these pathological inflammatory genes suggesting reduced transcriptional activation of pathological target genes by these crucial TFs. p300/CBP has been reported to deposit H3K27ac/H3K18ac to 'relax' chromatin superstructure and facilitate recruitment of basal transcription machinery to enable transcription 31. The underlying mechanisms leading to the decreased H3K27ac/H3K18ac deposition around the TF binding region remains to be defined. One possibility is that A-485 inhibits p300/CBP HAT activity and p300/CBP-catalyzed H3K27ac/H3K18ac at the TF-binding region. Another possibility is A-485 might inhibit TFs interaction with p300/CBP and reduced p300 recruitment on TF binding site. Furthermore, following A-485 administration *in vivo*, pro-inflammatory cytokine-mediated activation of NF-κB, MAPK, and NLRP3 signaling pathways and chemokine-mediated recruitment of inflammatory cells were decreased. Our data are consistent with the previous studies reporting the regulation of macrophage inflammatory responses by p300 45, 46. Regulation of a broad set of pathological genes is a crucial and distinguish feature of epigenetic inhibitor, as exemplified by a recent study showing that BET inhibitors could be used to treat heart failure by specifically suppressing innate inflammatory and pro-fibrotic transcriptional networks 47. In the present study, we revealed a global mechanism underlying the anti-inflammatory effects of targeted inhibition of p300 and also examined the *in vivo* effects.

Liver protectants and anti-inflammatory medications are the cornerstones of current therapies for ALI; however, an unchecked inflammatory response limits their effectiveness. Novel therapeutic approaches aimed to target macrophages, one of the pathogenic drivers of inflammation, by inhibiting macrophage activation, polarization, and leukocyte recruitment. We noticed that A-485 treatment influenced these processes. First, as discussed above, A-485 repressed macrophage activation and LPS-induced M1 polarization. Second, A-485 treatment inhibited the expression of *Ccl2*, *Ccl3* and *Ccl5*, chemokines which propagate further inflammatory cell infiltration, leading to a vicious cycle of hyper-inflammation and releasing distress signals in the microenvironment 12. Several pharmacological strategies interfering chemokine signaling, including antibodies against chemokines/receptors or the receptor antagonists, which prevent chemokine binding, are being tested in clinical trials to define their efficacy in liver diseases 48-52. The multiple inhibitory effects on macrophage activation, chemokine signaling, and macrophage polarization exerted by A-485 suggested its potential use as a protective therapeutic strategy for ALI treatment by targeting macrophages.

In summary, using a high-throughput screening assay, we identified A-485 as a potent inhibitor of macrophage activation and pro-inflammatory gene expression *in vitro* and *in vivo*. A-485, due to its potent anti-inflammatory ability, decreased the pathogenic inflammatory cascade thereby significantly improving survival rate as well as liver tissue structure and function in mice suffered ALI. RNA sequencing followed by KEGG pathway analysis revealed that genes affected by A-485 were also involved in rheumatoid arthritis and inflammatory bowel disease. We believe that p300/CBP inhibitor, A-485, can relieve inflammatory response by inhibiting macrophage function and might serve as a potential candidate for the treatment of ALI as well as other inflammatory diseases. Our work demonstrated the efficacy of an epigenome-based therapeutic approach that targets inflammatory state transition at the level of chromatin. Since the activated macrophages are a main pathogenic driver contributing to progression in many autoimmune diseases, such as rheumatoid arthritis, inflammatory bowel disease, colitis, multiple sclerosis, and atherosclerosis, A-485 might also have therapeutic potential for the treatment of these diseases.

## Supplementary Material

Supplementary figures and tables.

## Abbreviations

ALI: acute liver injury
GalN: D-galactosamine
LPS: lipopolysaccharide
MAPK: mitogen-activated protein kinase
NF-kappaB: the nuclear factor-kappaB
TLRs: toll-like receptors
TUNEL: terminal deoxynucleotidyl transferase dUTP nick-end labeling
