# Supplementary material for: p300/CBP inhibitor A-485 alleviates acute liver injury by regulating macrophage activation and polarization
Source: Theranostics. 2019 Oct 22;9(26):8344–61. doi: 10.7150/thno.30707 (PMC6857059; doi:10.7150/thno.30707)
Supplement: Supplementary file 1 — Supplementary figures and tables. [file thnov09p8344s1.pdf]

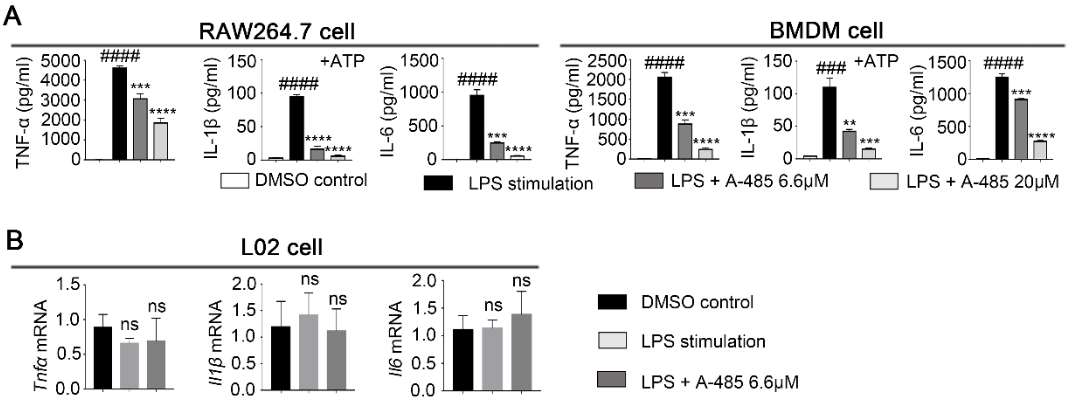

Figure S1. Macrophages played a leading role in the response of LPS and are major targets of A-485. (A) The RAW264.7 and BMDM cells were treated with LPS and A-485 for 4 h. The TNF-α, IL-1β and IL-6 concentrations in culture supernatants were determined by ELISA. **(B)** The L02 hepatocyte cells were treated with LPS and A-485 for 4 h, after which the *Tnfα*, *Il1β* and *Il6* mRNAs were quantified by RT-PCR analysis. (n=3) Data are shown as mean ± SD. ***P*<0.01, ****P*<0.001 and *****P*<0.0001 vs LPS group, ns *P*>0.05, ###*P*<0.001, ####*P*<0.0001 vs control group.

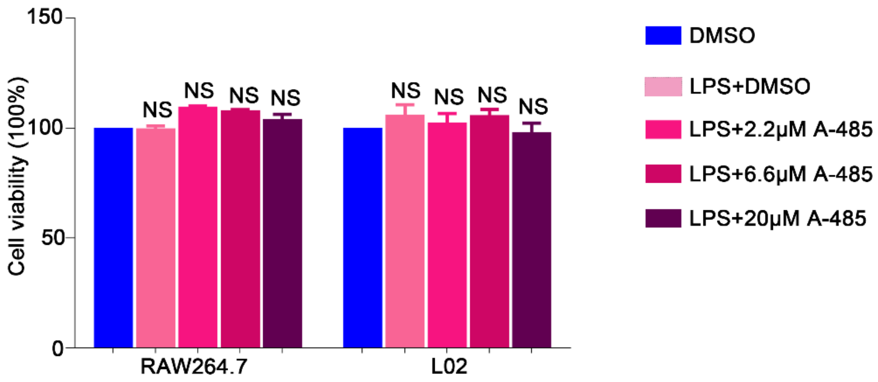

Figure S2. A-485's effects on cell viability. The viability of RAW264.7 and L02 cells after treated with various concentrations of A-485 for 24 h. ns *P*>0.05 vs control group.

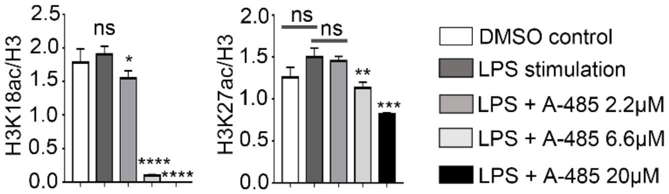

Figure S3. Quantifications of western blots in Figure 2C. The acetylation of H3K27 and H3K18 were reduced by A-485 treatment in concentration-dependent manner.

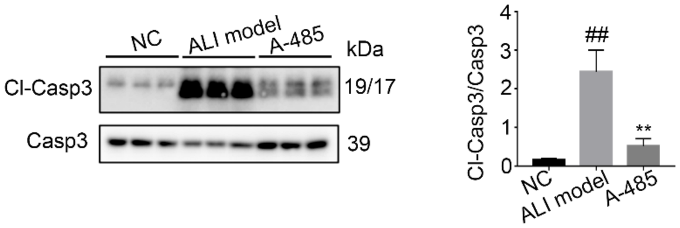

Figure S4. Western blots and quantification of in Caspase3 cleavage in the liver tissue. Western blot analysis showed the activation of Caspase3 was hindered after A-485 treatment and the quantified data was showed.

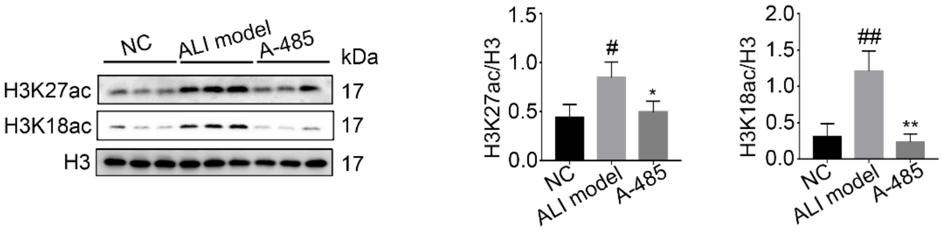

Figure S5. Western blot detected the acetylation level of p300/CBP substrates. A-485 specifically inhibited p300/CBP catalyzed acetylation of histone H3 lysine 27 (H3K27) and lysine 18 (H3K18) sites in vivo and the quantified data was showed.

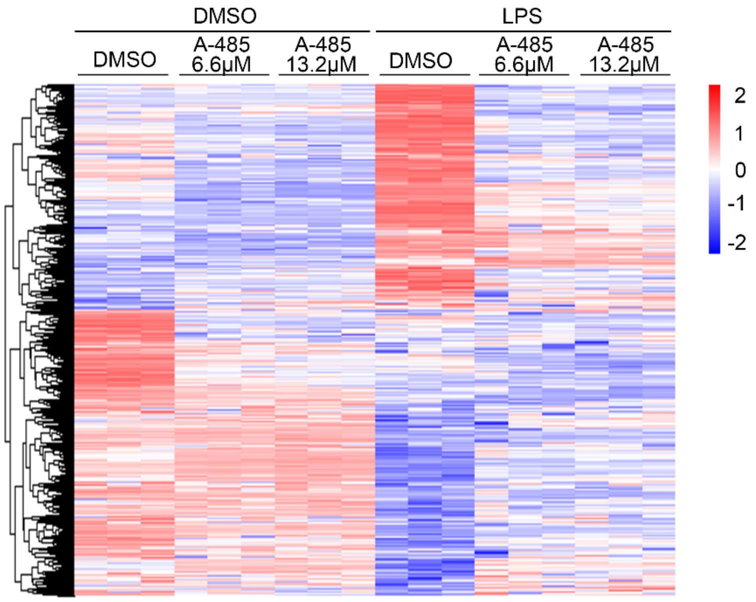

Figure S6. The heat map of all groups. Heat map showed gene expression profiles in DMSO control group, A-485 6.6μM control group, A-485 13.2μM control group, LPS stimulation + DMSO group, LPS stimulation + A-485 6.6μM group, LPS stimulation + A-485 13.2μM group.

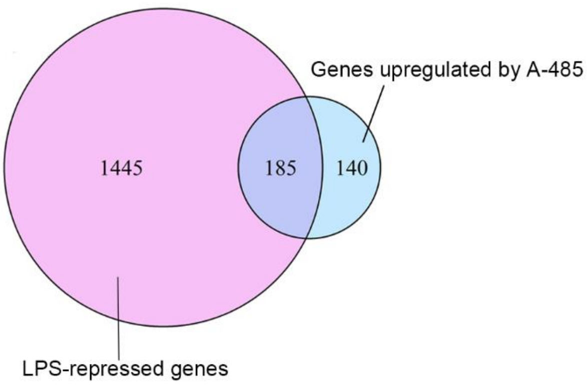

Figure S7. The contribution of A-485 to relieving LPS-induced repression is relatively marginal. Venn diagram showed the overlap between the gene set of LPS-repressed genes and the gene set of genes up-regulated by A-485 (13.2μM).

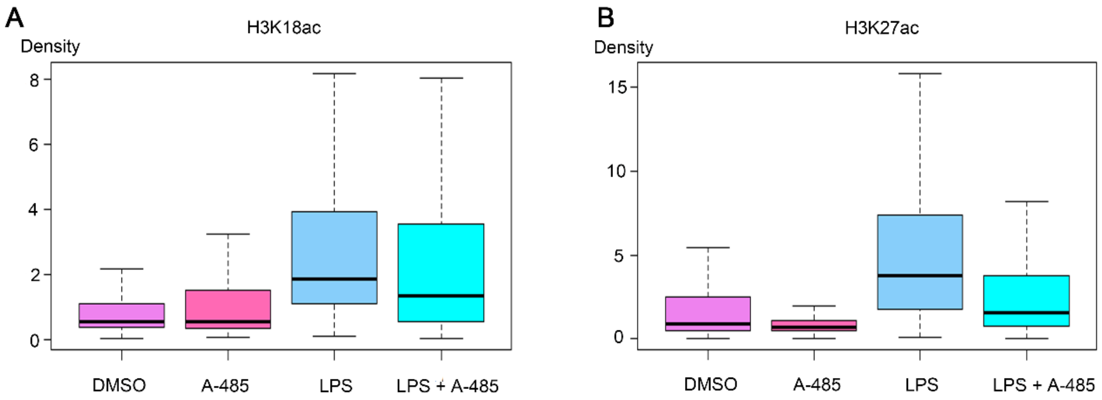

Figure S8. Boxplot to show H3K18ac and H3K27ac density in each group. A-485 treatment reduced H3K27ac **(B)** notably while H3K18ac **(A)** to a much less extent.

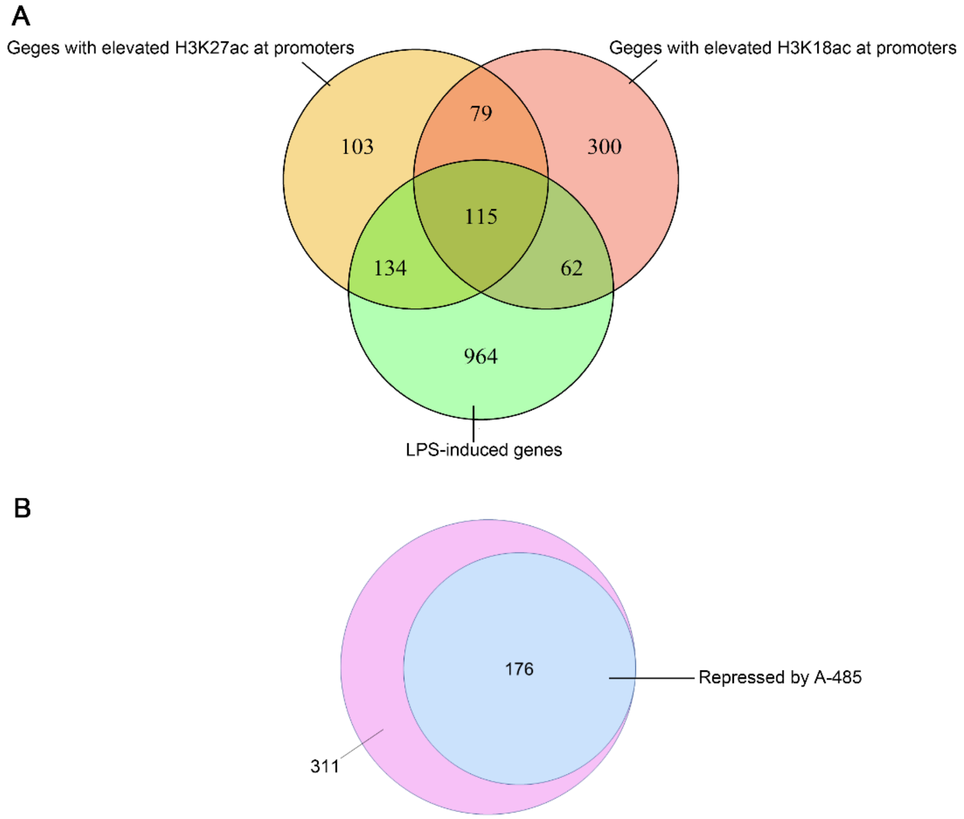

Figure S9. Integrative ChIP-Seq and transcriptome analysis indentified a specific set of genes directly regulated by A-485 on both transcriptional and epigenetic level. (A) Venn diagram showed the overlap between the gene set of LPS-induced up-regulated genes (1275 genes; green), the gene set of genes with elevated H3K18ac upon LPS stimulation (red), and the gene set of genes with elevated H3K27ac upon LPS stimulation (yellow). 311 genes in the overlap (134+115+62=311) showed correspondently elevated H3K27ac or H3K18ac at their promotors upon LPS stimulation. **(B)** Among the 311 genes, 176 genes are represses by A-485 on transcriptional level and shows correspondently decreased H3K27ac or H3K18ac.

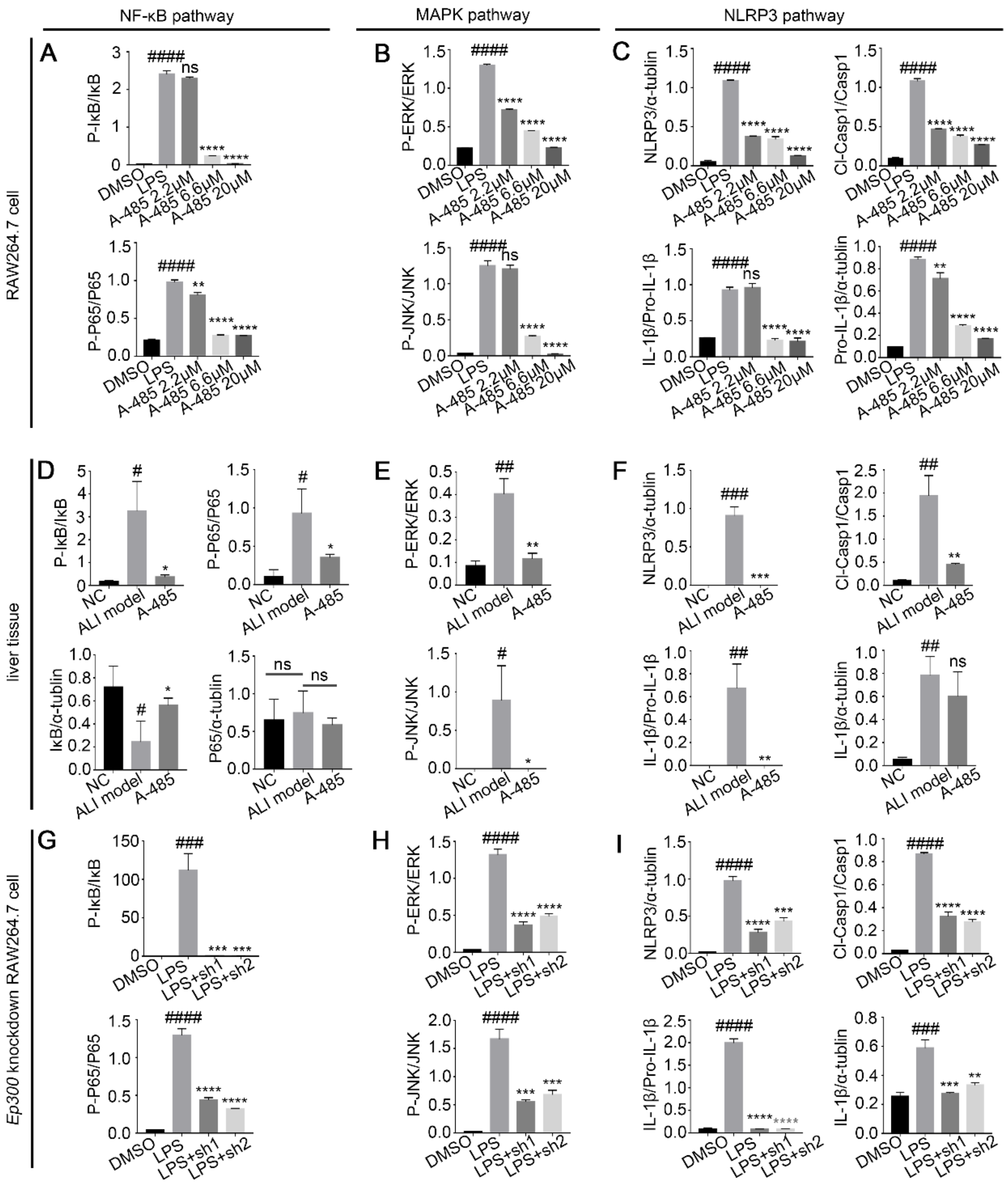

Figure S10. Quantifications of the western blots in figure 7A in manuscript.

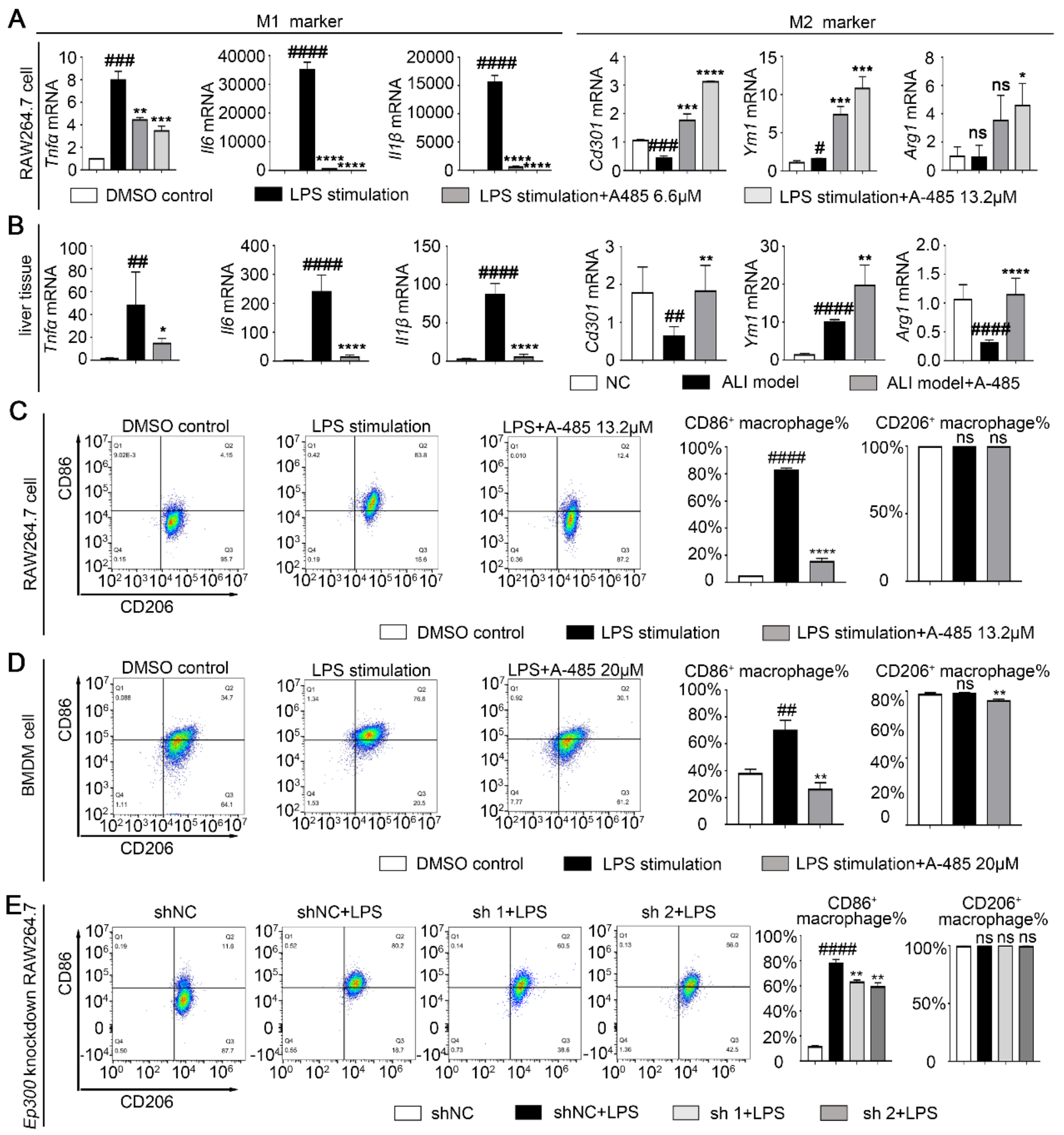

Figure S11. A-485 treatment suppressed M1-phenotype macrophage polarization. (A-B) RT-qPCR analysis of mRNA expression of indicated M1 and M2 marker genes in LPS-challenged RAW264.7 cells untreated or treated with A-485 in indicated concentrations (n=3) **(A)** or in liver tissue (n=6) **(B)**. **(C-E)** Flow cytometry analysis of RAW264.7 cells **(C)**, BMDM cells **(D)** and *Ep300* knockdown RAW264.7 cells **(E)** stimulated with LPS and treated or untreated with A-485. M1 marker CD86 and M2 marker CD206 were used for flow cytometry analysis. Dot blots **(C-E, left)** show that induction of the M1 marker by LPS is significantly reduced by A-485, while the M2 marker is not significantly affected. Graphs **(C–E, right)** show quantitative statistical analysis of M1 marker and M2 marker in cells (n=3). Data are shown as mean ± SD. *P<0.05 and **P<0.01, ***P<0.001 and ****P<0.0001 vs LPS group, ns P>0.05, #P<0.05, ##P<0.01, ### P<0.001 and ####P<0.0001 vs control group.

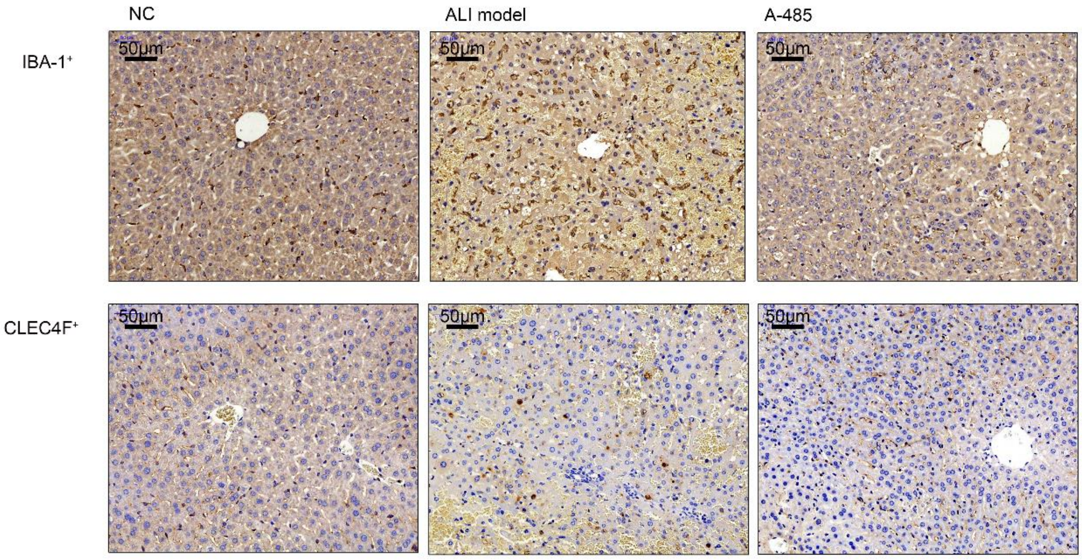

Figure S12. Immunohistochemistry staining of IBA-1^{+} and CLEC4F^{+} cells in liver tissue.

Table S1. shRNA sequences used for *Ep300* gene knockdown

shRNAs	Oligonucleotides(5'-3')
**sh*Ep300*#1**	CGC GAA TGA CAA CAC AGA TTT CTC GAG AAA TCT GTG TTG TCA TTC GCG TTTT
**sh*Ep300*#2**	TAA CTC TGG CCA TAG CTT AAT CTC GAG ATT AAG CTA TGG CCA GAG TTA TTTT

Table S2. Primers used for real-time PCR studies

Genes		Oligonucleotides (5'-3')
Ccl5	Forward	GTG CCC ACG TCA AGG AGT AT
	Reverse	TTC TCT GGG TTG GCA CAC AC
Ep300	Forward	GGG ACT AAC CAA TGG TGG TG
	Reverse	ATT GGG AGA AGT CAA GCC TG
Gapdh	Forward	GGT GAA GGT CGG TGT GAA CGG A
	Reverse	CCA AAG TTG TCA TGG ATG ACC TTG G
Tnfα	Forward	GCA ACT GCT GCA CGA AAT C
	Reverse	CTG CTT GTC CTC TGC CCA C
Il1β	Forward	TCT TTG AAG TTG ACG GAC CC
	Reverse	TGA GTG ATA CTG CCT GCC TG
Il6	Forward	AGT TGC CTT CTT GGG ACT GA
	Reverse	TTC TGC AAG TGC ATC ATC GT
Ccl20	Forward	TTG CTC CTG GCT GCT TTG AT
	Reverse	GCC GTG TGA AGC CCA CAA TA
Ccl2	Forward	TAA AAA CCT GGA TCG GAA CCA AA
	Reverse	GCA TTA GCT TCA GAT TTA CGG GT
Il1α	Forward	AGT ATC AGC AAC GTC AAG CAA
	Reverse	TCC AGA TCA TGG GTT ATG GAC TG
Cd301	Forward	AAA GGC TTT AAG AAC TGG GC
	Reverse	TAG TGA TGT GGG CAC AGT C
Ym1	Forward	TAC TAT GAG GCT CAG TGG C
	Reverse	ACA GAA AGA ACC ACT GAA GTC
Arg1	Forward	GAA CTG AAA GGA AAG TTC CCA
	Reverse	AAT GTA CAC GAT GTC TTT GGC

Table S3. Antibodies used for Western Blot studies

Reagent	Source	Catalog
**H3K18ac**	Cell Signaling Technology	Cat# 9675

**H3K27ac**	Cell Signaling Technology	Cat# 8173
**H3**	Cell Signaling Technology	Cat# 14269
**p-IKK**	Cell Signaling Technology	Cat# 2697
**IKK**	Cell Signaling Technology	Cat#2678
**p-IκB**	Cell Signaling Technology	Cat# 2859
**IκB**	Cell Signaling Technology	Cat# 4814
**p-p65**	Cell Signaling Technology	Cat# 3033
**p65**	proteintech	Cat# 10745-I-AP
**p-ERK**	Servicebio	Cat# 180124
**ERK**	Servicebio	Cat# 180291
**p-JNK**	Cell Signaling Technology	Cat# 4668
**JNK**	Cell Signaling Technology	Cat# 9252
**Casp3**	Cell Signaling Technology	Cat# 9662
**Cl-Casp3**	Cell Signaling Technology	Cat# 9664
**Cl-Casp1**	Cell Signaling Technology	Cat# 89332
**Casp1**	Santa Cruz Biotechnology	Cat# 56036
**IL-1β**	Cell Signaling Technology	Cat# 63124
**Pre-IL-1β**	Cell Signaling Technology	Cat# 12242
**NLRP3**	Cell Signaling Technology	Cat# 15101
**α-Tublin**	Cell Signaling Technology	Cat# 3873

Table S4. The compound library and their targets.

**Targets**		**Probes**
SYMD2		LLY-507
		BAY-598
KDM5		KDM5-IN-1
G9a		UNC0638
SETD7		PFI-2 (hydrochloride)
Sirtuin	Sirtuin1,2	Tenovin-1
	Sirtuin1	EX-527
	Sirtuin2	Thiomyristoyl
	Sirtuin3	3-TYP
LSD1		GSK2879552
		Tranylcypromine (hemisulfate)
DOT1L		EPZ004777
		EPZ-5676
EZH2		GSK126
		CPI-1205
		EPZ-6438
WDR5		WDR5-0103
WDR5/MLL		MM-102 (trifluoroacetate)
		OICR-9429

<table>
<tr><td colspan="2">CREB</td><td>666-15</td></tr>
<tr><td colspan="2" rowspan="2">CBP/p300 bromodomain</td><td>CPI-637</td></tr>
<tr><td>SGC-CBP30</td></tr>
<tr><td colspan="2">CBP/p300 HAT domain</td><td>A-485</td></tr>
<tr><td rowspan="6">HDAC</td><td>HDAC1,3,6</td><td>Resminostat (hydrochloride)</td></tr>
<tr><td>HDAC1,2</td><td>Romidepsin</td></tr>
<tr><td>HDAC1,3</td><td>Givinostat (hydrochloride monohydrate)</td></tr>
<tr><td>HDAC1，HDAC2，HDAC3 (Class I)，HDAC7 (Class II) ，Class IV (HDAC11)</td><td>vorinostat; SAHA</td></tr>
<tr><td rowspan="2">pan-HDAC</td><td>PCI-24781</td></tr>
<tr><td>Belinostat</td></tr>
<tr><td rowspan="6">PRMTs</td><td>PRMT3</td><td>SGC707</td></tr>
<tr><td>Type I PRMTs</td><td>MS023</td></tr>
<tr><td>PRMT4，6</td><td>MS049</td></tr>
<tr><td>PRMT4</td><td>SGC2085</td></tr>
<tr><td>PRMT6</td><td>EPZ020411 (hydrochloride)</td></tr>
<tr><td>PRMT1,3,4,6,8</td><td>DCPR049_12</td></tr>
<tr><td colspan="2">Menin-MLL</td><td>MI-538</td></tr>
<tr><td colspan="2">CBX7-H3</td><td>UNC3866</td></tr>
<tr><td colspan="2">BMI-1</td><td>PTC-209</td></tr>
<tr><td colspan="2" rowspan="2">EED</td><td>EED226</td></tr>
<tr><td>A-395</td></tr>
<tr><td colspan="2">20S proteasome</td><td>Bortezomib</td></tr>
<tr><td colspan="2">topoisomerase Ⅱ</td><td>Etoposide</td></tr>
<tr><td colspan="2">Tigger apotosis</td><td>Elesclomol</td></tr>
<tr><td colspan="2">proteasome</td><td>Carfilzomib</td></tr>
<tr><td colspan="2">topoisomerase I</td><td>Irinotecan</td></tr>
<tr><td colspan="2">HSP90</td><td>NVP-AUY922</td></tr>
<tr><td colspan="2">IRF3，HSP90</td><td>Geldanamycin</td></tr>
<tr><td colspan="2">PARP</td><td>Rucaparib (phosphate)</td></tr>
<tr><td colspan="2">PARP1,2</td><td>Olaparib</td></tr>
<tr><td colspan="2">PARP1</td><td>MK-4827</td></tr>
</table>

Table S5. The full list of hub genes.

**Gene**	**Degree**
Il1β	8
Il6	7
Ccl12	6
Ccl2	6
Ifng	6

Tgfb2	5
Ccl20	5
Il1α	5
Csf2	5
Csf1	5
H2-Ab1	4
Fos	4
Cxcl5	4
Cxcl2	4
Ccl5	4
Il17α	4
Cxcl1	4
Csf3	4

Table S6. Top 30 most enriched LPS-affected motifs associated with increased H3K27ac. Top 30 motifs associated with increased H3K27ac after LPS stimulation were shown, and A-485 affected motifs (with decreased H3K27ac after A-485 treatment) among them are highlighted in blue.

Name	Rank	Motif	P-value
Atf3 (bZIP)	1	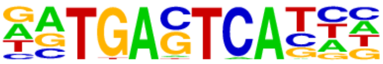	1.00E-99
BATF (bZIP)	2	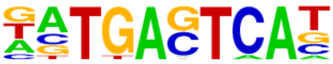	1.00E-99
Fra1 (bZIP)	3	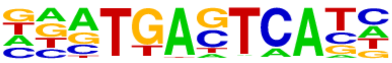	1.00E-94
AP-1 (bZIP)	4	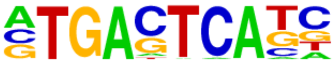	1.00E-90
Fosl2 (bZIP)	5	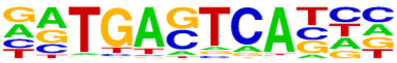	1.00E-77
Atf4 (bZIP)	6	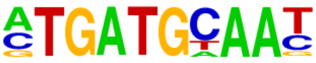	1.00E-64
Jun-AP1 (bZIP)	7	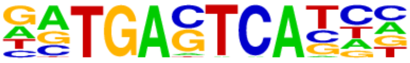	1.00E-63
Chop (bZIP)	8	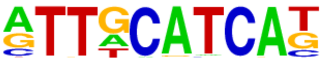	1.00E-62
ETS1 (ETS)	9	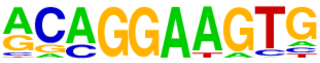	1.00E-40
ERG (ETS)	10	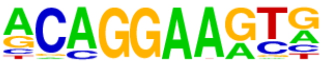	1.00E-40
Atf7 (bZIP)	11	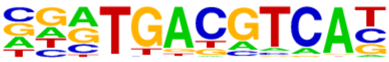	1.00E-38
Fli1 (ETS)	12	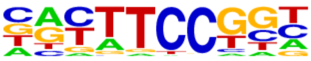	1.00E-37

ETV1 (ETS)	13	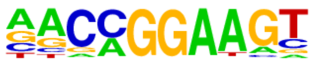	1.00E-36
Atf2 (bZIP)	14	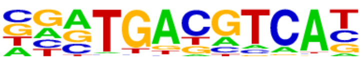	1.00E-35
Atf1 (bZIP)	15	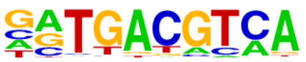	1.00E-35
c-Jun-CRE (bZIP)	16	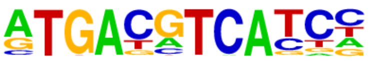	1.00E-34
ISRE (IRF)	17	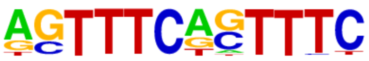	1.00E-32
IRF1 (IRF)	18	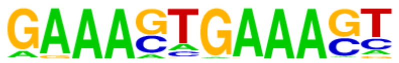	1.00E-31
Ets1-distal (ETS)	19	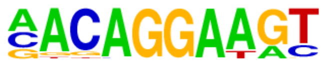	1.00E-31
Bach2 (bZIP)	20	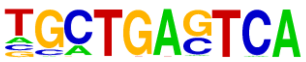	1.00E-29
JunD (bZIP)	21	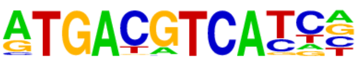	1.00E-29
IRF2 (IRF)	22	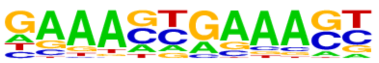	1.00E-29
CEBP:AP1 (bZIP)	23	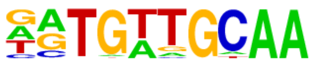	1.00E-28
NFkB-p65 (RHD)	24	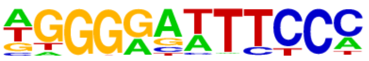	1.00E-26
GABPA (ETS)	25	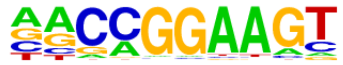	1.00E-26
ELF5 (ETS)	26	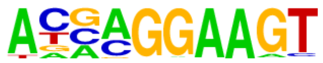	1.00E-24
EWS:ERG-fusion (ETS)	27	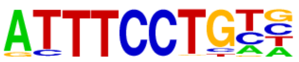	1.00E-24
NFkB-p65-Rel (RHD	28	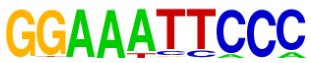	1.00E-23
EHF (ETS)	29	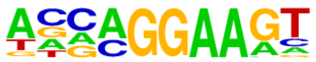	1.00E-22
PU.1-IRF (ETS:IRF)	30	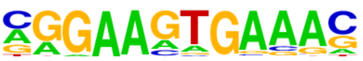	1.00E-18

Table S7 Top 30 most enriched LPS-affected motifs associated with increased H3K18ac. Top 30 motifs associated with increased H3K18ac after LPS stimulation were shown, and A-485 affected motifs (with decreased H3K18ac after A-485 treatment) among them are highlighted in blue.

Name	Rank	Motif	P-value
AP-1 (bZIP)	1	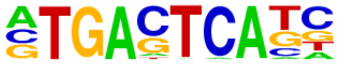	1e-136
BATF (bZIP)	2	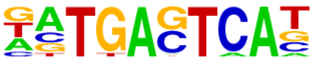	1e-136
Atf3 (bZIP)	3	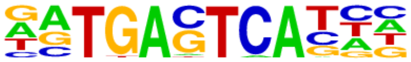	1e-130
Fra1 (bZIP)	4	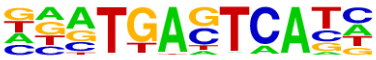	1e-119
Fosl2 (bZIP)	5	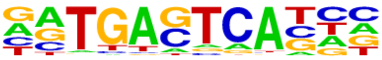	1e-101
Atf4 (bZIP)	6	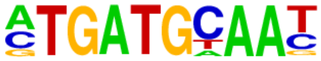	1e-98
Chop (bZIP)	7	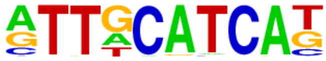	1e-92
Jun-AP1 (bZIP)	8	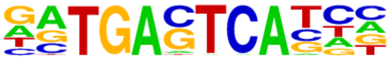	1e-77
Atf1 (bZIP)	9	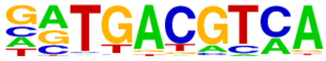	1e-75
Atf7 (bZIP)	10	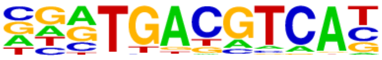	1e-74
c-Jun-CRE (bZIP)	11	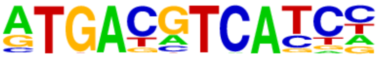	1e-65
Atf2 (bZIP)	12	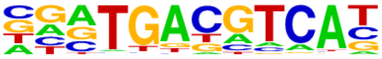	1e-62
JunD (bZIP)	13	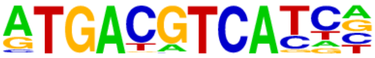	1e-51
Bach2 (bZIP)	14	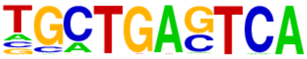	1e-50
ETS1 (ETS)	15	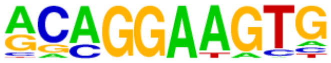	1e-49
CEBP:AP1 (bZIP)	16	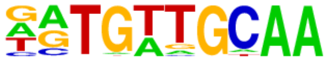	1e-45
ERG (ETS)	17	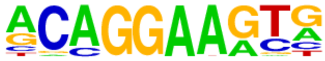	1e-43
ETV1 (ETS)	18	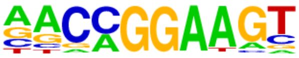	1e-43

Fli1 (ETS)	19	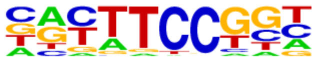	1e-41
Ets1-distal (ETS)	20	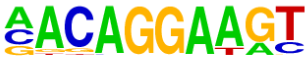	1e-35
GABPA (ETS)	21	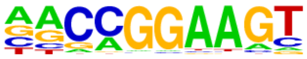	1e-35
EWS:ERG-fusio (ETS)	22	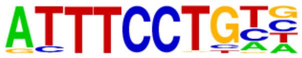	1e-31
EHF (ETS)	23	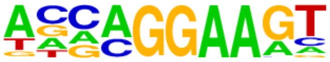	1e-30
EWS:FLI1-fusion (ETS)	24	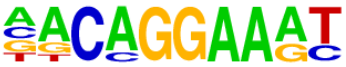	1e-29
Elk1 (ETS)	25	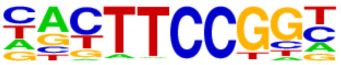	1e-27
ISRE (IRF)	26	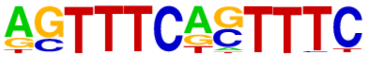	1e-25
Elk4 (ETS)	27	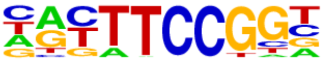	1e-23
NFkB-p65 (RHD)	28	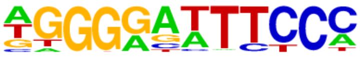	1e-23
IRF2 (IRF)	29	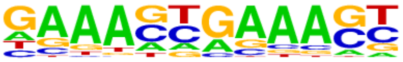	1e-23
IRF1 (IRF)	30	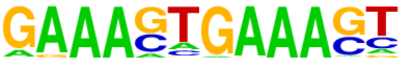	1e-20
